# Inhibition of the H_V_1 voltage-gated proton channel compromises the viability of human polarized macrophages in a polarization- and ceramide-dependent manner

**DOI:** 10.3389/fimmu.2024.1487578

**Published:** 2024-12-17

**Authors:** Tamas Kovacs, Bence Cs. Szabo, Rosemary Chandrakanthi Kothalawala, Virag Szekelyhidi, Peter Nagy, Zoltan Varga, Gyorgy Panyi, Florina Zakany

**Affiliations:** Department of Biophysics and Cell Biology, Faculty of Medicine, University of Debrecen, Debrecen, Hungary

**Keywords:** H_V_1, cell viability, M1 macrophages, M2 macrophages, polarization, ceramide, pH regulation, acid sphingomyelinase

## Abstract

The human voltage-gated proton channel (H_V_1) provides an efficient proton extrusion pathway from the cytoplasm contributing to the intracellular pH regulation and the oxidative burst. Although its pharmacological inhibition was previously shown to induce cell death in various cell types, no such effects have been examined in polarized macrophages albeit H_V_1 was suggested to play important roles in these cells. This study highlights that 5-chloro-2-guanidinobenzimidazole (ClGBI), the most widely applied H_V_1 inhibitor, reduces the viability of human THP-1-derived polarized macrophages at biologically relevant doses with M1 macrophages being the most, and M2 cells the least sensitive to this compound. ClGBI may exert this effect principally by blocking H_V_1 since the sensitivity of polarized macrophages correlates well with their H_V_1 expression levels; inhibitors of other macrophage ion channels that may be susceptible for off-target ClGBI effects cause no viability reductions; and Zn^2+^, another non-specific H_V_1 blocker, exerts similar effects. As a potential mechanism behind the ClGBI-induced cell death, we identify a complex pH dysregulation involving acidification of the cytoplasm and alkalinization of the lysosomes, which eventually result in membrane ceramide accumulation. Furthermore, ClGBI effects are alleviated by ARC39, a selective acid sphingomyelinase inhibitor supporting the unequivocal significance of ceramide accumulation in the process. Altogether, our results suggest that H_V_1 inhibition leads to cellular toxicity in polarized macrophages in a polarization-dependent manner, which occurs due to a pH dysregulation and concomitant ceramide overproduction mainly depending on the activity of acid sphingomyelinase. The reduced macrophage viability and plausible concomitant changes in homeostatic M1-M2 balance could contribute to both the therapeutic and potential side effects of H_V_1 inhibitors that show great promise in the treatment of neuroinflammation and malignant diseases.

## 1 Introduction

The human voltage-gated proton channel (H_V_1) is built up by four transmembrane helical segments that act as a voltage-sensor domain and a physiologically outwardly directed proton-conductive pathway in the cell membrane. H_V_1 is regulated by the membrane potential and pH, activating at depolarizing voltages and during cytosolic acidification (1, 2). While other voltage-gated channels are composed of four voltage-sensor domains controlling one central permeation pore domain, H_V_1 has a unique dimer structure lacking a conventional pore with each subunit having its own proton permeation pathway localized in the voltage-sensor domain and one gate controlled by one voltage sensor (3). H_V_1 is expressed in a wide variety of human cell types in which the channel is mainly involved in two major cellular functions. First, a H_V_1-mediated proton extrusion from the cytoplasm provides a charge compensation for the transmembrane electron transfer occurring due to the activity of the NADPH oxidase and is thus required for a sustained production of reactive oxygen species (ROS). This counterbalancing activity has been linked to the respiratory burst and bacterial clearance of neutrophil granulocytes (4–6), antibody production of B lymphocytes (7), transport-mediated CO_2_ disposal in the heart (8), immunosuppressive activity of myeloid-derived suppressor cells (9) and various effector functions of T lymphocytes (10). While such regulation is essential for these physiological functions, a H_V_1-enabled ROS overproduction can also contribute to activation and proinflammatory cytokine production of microglia in neuroinflammation (11). Second, the proton efflux mediated by H_V_1 efficiently modulates intracellular pH for appropriate sperm activation, motility and capacitation (6, 12, 13) or migration and mineral matrix production of chorion-derived mesenchymal stem cells (14). However, H_V_1 can also relieve extensive metabolic acidosis that frequently arises during the rapid proliferation of tumor cells and, therefore, its overexpression frequently observed in malignant neoplasms facilitates tumor growth (9).

Although various peptide and small-molecule compounds have been described thus far to inhibit the conductive function of H_V_1 (6, 15–19), guanidine-containing compounds, particularly 5-chloro-2-guanidinobenzimidazole (ClGBI), are still the most widely applied for this purpose (8, 9, 13, 14, 20–25). Due to the mitigation of ROS overproduction, an inhibition of H_V_1 was proposed to show great promise in acute lung injury (26), ischemic stroke (27, 28), multiple sclerosis (29, 30), brain and spinal cord injury (31–33), and peripheral neuropathic and inflammatory pain (18, 34). Furthermore, by eliminating the protection from proliferation-associated metabolic acidosis, a H_V_1 block was proposed to favorably reduce the growth rate of various malignant tumors such as breast cancer (25, 35, 36), colorectal carcinoma (37), glioblastoma multiforme (38) or leukemia (15, 39). The pH dysregulation associated with blocking H_V_1 conductivity can lead to cell death by triggering apoptotic processes, which may thus contribute to both the intended and adverse effects of the compounds. Consistently, a pharmacological H_V_1 inhibition was previously shown to compromise the viability of various cell types including Jurkat T lymphocytes (20), chorion-derived mesenchymal stem cells (14), activated mouse microglia (23), myeloid-derived suppressor cells (9), breast cancer (25) and glioblastoma multiforme cell lines (38).

Macrophages, essential cellular components of the innate immune system, are plastic and heterogeneous cells whose differentiation and activation are distinctively elicited by a large variety of stimuli. In general, macrophages are classified into M1, or classically activated, and M2, or alternatively activated subsets. M1 macrophages activated by Th1 cytokines, such as interferon-γ, and Toll-like receptor ligands, e.g., bacterial lipopolysaccharide (LPS), produce proinflammatory cytokines and mediators, and participate in defense mechanisms against pathogens and tumors, while M2 macrophages induced by Th2 or anti-inflammatory cytokines, such as interleukin-4 (IL-4), interleukin-13 (IL-13) or interleukin-10 (IL-10), are rather involved in the resolution of inflammation, scavenging apoptotic cells and debris, tissue remodeling and immune regulation (40, 41). Although this dichotomous classification is convenient, it is largely oversimplified since M2 macrophages can be further divided into distinct functional subpopulations. Therefore, M1 and M2 phenotypes rather represent extremes of a continuum of functional states, and these phenotypes are dynamically interconvertible through reprogramming depending on microenvironmental signals (42–44). Nevertheless, the M1-M2 classification is still an operationally useful framework for didactic reasons and because dysregulated macrophage polarization is often associated with the occurrence and progression of various diseases. An imbalance in macrophage polarization with proinflammatory M1 dominance was linked to a consequently enhanced inflammation in atherosclerosis, obesity and metabolic disorder, and autoimmune diseases such as Crohn’s disease, rheumatoid arthritis, multiple sclerosis or autoimmune hepatitis. On the contrary, an M2 preponderance can contribute to allergic airway inflammation and asthma, and a shift from tumor-infiltrating M1 macrophages to a predominantly M2-like tumor-associated macrophage phenotype is associated with immune evasion of malignant tumors. Hence, altering the balance of macrophage polarization is considered as a potential therapeutic target in these disorders and, therefore, understanding the factors regulating polarization and the role of certain proteins in differentially activated macrophages carries substantial biomedical relevance (43, 45). H_V_1 expression was previously described in murine and human, including THP-1-derived macrophages as well (46, 47). In these cells, the proton efflux mediated by H_V_1 was subsequently shown to play important roles in the sustained ROS production (48), proper phagosomal acidification and pH oscillations (49, 50), and protection from intracellular fungal pathogens (51). However, the potential effects of blocking the H_V_1 channel on the viability of polarized macrophages have not been thoroughly tested yet in spite of the fact that a reduced viability and plausible changes in homeostatic M1-M2 balance could largely alter both macrophage-related physiological and pathophysiological processes.

As mentioned above, intracellular pH dysregulation caused by H_V_1 inhibition is often associated with triggering cell death processes (9, 20, 23, 25, 38). However, the molecular details of this connection have not been elucidated yet. Membrane ceramides comprise a special class of sphingolipids, which are characterized by unique membrane biophysical properties due to their extreme hydrophobicity and compactness resulting from a very small hydrophilic headgroup (52–54). While ceramides are generally present normally at minuscule levels in the cell membrane, various stress stimuli including tumor necrosis factor α (TNFα), ionizing radiation and chemotherapeutic drugs result in their accumulation, which in turn activates different forms of cell death such as necrosis or apoptosis. Accordingly, the level of membrane ceramides is strictly regulated by a highly complex interconnected network of enzymes involved in their production and degradation. Ceramides typically accumulate through *de novo* synthesis, which mainly depends on the activity of the rate-limiting enzyme, serine palmitoyltransferase, or degradation of the ubiquitous sphingomyelin by neutral or acidic sphingomyelinase (55–57). Given that activities of these enzymes strongly depend on pH (58–60), it is tempting to speculate that complex pH alterations associated with H_V_1 inhibition may lead to cell death through ceramide overproduction.

In this study, we show that ClGBI, the most widely applied inhibitor of H_V_1, dose-dependently reduces the viability of human THP-1-derived polarized macrophages at biologically relevant doses and the sensitivity of the distinctly polarized macrophages differ with M1 cells being the most prone, and M2 macrophages the least sensitive to the compound. Through obtaining similar results with Zn^2+^, another H_V_1 blocker, and demonstrating the lack of notable effects of blockers of other ion channels that are relevant in macrophages and possible subjects of off-target ClGBI inhibition, we also provide experimental evidence for this ClGBI-induced effect indeed being mediated predominantly through H_V_1 inhibition. In addition, we show that the compromised viability occurs due to a complex pH dysregulation involving both the cytoplasmic and lysosomal compartments, and it is accompanied by elevations in membrane ceramide levels. Furthermore, supporting the intrinsic role of ceramide in the H_V_1 block-induced compromised viability of macrophages, we demonstrate that the effects of ClGBI can be alleviated by ARC39, a compound that selectively inhibits acid sphingomyelinase-mediated ceramide production.

## 2 Materials and methods

### 2.1 Cell culture, differentiation and polarization of macrophages

The human acute monocytic leukemia-derived cell line THP-1 was obtained from the American Type Culture Collection (Manassas, VA) and cultivated according to its specifications. THP-1 cells were differentiated into macrophages for 24 h by phorbol 12-myristate 13-acetate (PMA; Sigma-Aldrich, St. Louis, MO) at a final concentration of 10 ng/ml to achieve sufficient differentiation and minimize unspecific gene expression changes (61, 62), which was followed by a resting period of 24 h in the absence of PMA to ensure the M0 phenotype of the produced macrophages (61, 63). The differentiated M0 macrophages were subsequently polarized for 24-48 h into classical M1 macrophages with 100 ng/ml lipopolysaccharide (LPS; E. coli O111:B4 ultrapure, Sigma-Aldrich) plus 20 ng/ml interferon-γ (IFN-γ; Thermo Fisher Scientific, Waltham, MA), or M2 macrophages using 20 ng/ml interleukin-4 (IL-4; Thermo Fisher Scientific) plus 20 ng/ml interleukin-13 (IL-13; Thermo Fisher Scientific), according to a widely applied protocol (64–67). All treatments were carried out at 37°C.

### 2.2 Analysis of plasma membrane expression of CD markers in polarized macrophages

Differentiated and polarized THP-1-derived macrophages grown in 6-well plates were washed and treated with accutase (Sigma-Aldrich) at room temperature for 5 min to gently detach adherent cells without compromising cell viability (68). After stopping the reaction with cell culture medium and washing, Fc receptors of cells were blocked for 20 min with human FcR blocking reagent (Miltenyi Biotec, Bergisch Gladbach, Germany) applied at 10 µg/ml. Subsequently, cells were labeled with one of the following antibodies: FITC-conjugated anti-CD64 (at a concentration of 4 µg/ml), Alexa Fluor 647-conjugated anti-CD71 (4 µg/ml), FITC-conjugated anti-CD80 (4 µg/ml), PE-Cy5-conjugated CD86 (1 µg/ml), PE-conjugated CD206 (0.5 µg/ml). All antibodies were obtained from Thermo Fisher and applied for 20 min at room temperature. After washing, the fluorescence intensity of individual cells was measured using a NovoCyte 3000RYB flow cytometer (ACEA Biosciences, San Diego, CA) with the following excitation wavelengths and emission filters, respectively: FITC – 488 nm and 530/30 nm; Alexa Fluor 647 – 640 nm and 660/20 nm; PE-Cy5 – 561 nm and 660/20 nm; PE – 561 nm and 586/20 nm. During data analysis carried out in FCS Express (De Novo Software, Los Angeles, CA), the average fluorescence intensity of at least 10,000 cells of normal morphology on FSC-SSC dot plots was calculated for each sample. The low extent of nonspecific binding of antibodies was supported by data obtained with isotope control antibodies FITC-conjugated Mouse IgG1 kappa Isotype Control, PE-Cy5-conjugated Mouse IgG1 kappa Isotype Control, PE-conjugated Mouse IgG1 kappa Isotype Control and Alexa Fluor 647-conjugated Mouse IgG2b kappa Isotype Control (all from Thermo Fisher) applied at identical experimental conditions (**Supplementary Figure 1**).

### 2.3 Examination of effects of ion channel inhibitors on the viability of polarized macrophages

THP-1 cells seeded into 24-well plates were differentiated into macrophages and polarized as above, and treated subsequently for 24 h with ion channel inhibitors supplemented into the culture media of cells. In the case of ClGBI (5-chloro-2-guanidinobenzimidazole; Sigma-Aldrich), a dilution series with concentrations ranging between 16 and 256 µM was used, while in other experiments cells were treated with 20 pM Vm24 scorpion toxin (Alomone Labs, Jerusalem, Israel), 20 nM TTX (tetrodotoxin; Alomone Labs), 1 mM TEA^+^ (tetraethylammonium; Sigma-Aldrich), or 1 mM (nominal) ZnCl_2_ (Sigma-Aldrich). For the investigation of potential protective effects of pretreatments with ceramide production inhibitors against ClGBI-induced toxicity, 1 µM myriocin (Sigma-Aldrich), 2 µM GW4869 (Sigma-Aldrich) or 5 µM ARC39 (Cayman Chemical, Ann Arbor, MI) was given to polarized M0, M1 and M2 macrophages 30 min before the application of ClGBI. For the determination of the fraction of viable cells calculated as *viable fraction = number of double negative cells/number of all cells*, as described previously (53, 69, 70), we collected the supernatant containing cells detached from the surface of the well, and pooled them with the adherent cells, which were detached by accutase. This cell suspension was labeled with Sytox Green Dead Cell Stain (Thermo Fisher Scientific) and Alexa Fluor 647-conjugated annexin V (Thermo Fisher Scientific) at dilutions of 1:1000 and 1:20, respectively, in annexin binding buffer for 15 minutes at room temperature. Fluorescence intensities of at least 10,000 individual cells per sample were subsequently measured using a NovoCyte 3000RYB flow cytometer. Sytox Green and Alexa Fluor 647 fluorophores were excited at 488 and 640 nm, respectively, and emitted intensities were measured using 530/30 and 660/20 emission filters, respectively. During data analysis, the fraction of Sytox Green and annexin V negative viable cells was calculated for each sample using FCS Express.

### 2.4 Determination of H_V_1 expression in polarized macrophages

Differentiated and polarized THP-1-derived macrophages grown in 12-well plates were washed and detached with accutase, which was followed by fixation in ice-cold methanol (Sigma-Aldrich) for 20 minutes. After permeabilization in 0.1% Triton X-100 (Sigma-Aldrich), cells were blocked with human FcR blocking reagent and 5% BSA for 30 minutes at room temperature. Subsequently, cells were labeled with anti-H_V_1 antibodies (PA5-24964, Thermo Fisher Scientific) for 60 minutes at room temperature at 5 µg/ml and, after washing, with Alexa Fluor 647-conjugated goat anti-rabbit IgG antibodies (Thermo Fisher Scientific) for 30 minutes at room temperature at a dilution of 1:500 (25). All steps were carried out in a solution containing 1% BSA and 0.1% Triton X-100. The fluorescence intensity of individual cells was measured with NovoCyte 3000RYB using excitation at 640 nm and a 660/20 emission band pass filter and average fluorescence intensities of at least 10,000 cells with a normal morphology gated on FSC-SSC plots were calculated in each sample in FCS Express. The low extent of nonspecific binding of the primary antibody was supported by data obtained with the isotope control antibody Rabbit IgG Isotype Control (Thermo Fisher) applied at identical experimental conditions (**Supplementary Figure 2**).

### 2.5 Investigation of time-dependent effects of ClGBI on the cytoplasmic pH of polarized macrophages

THP-1 cells seeded into 24-well plates were differentiated into macrophages and polarized as above, and treated subsequently for 1, 4 or 18 h with 50 or 100 µM ClGBI supplemented into the culture media of cells. In the last 30 min of incubation at 37°C, 5 µM pHrodo Red AM Intracellular pH Indicator (Thermo Fisher) was further added to cells (71). After washing and detachment of cells with accutase, the fluorescence intensity of individual cells was measured with NovoCyte 3000RYB using excitation at 561 nm and a 586/20 emission band pass filter and average fluorescence intensities of at least 10,000 cells with a normal morphology gated on FSC-SSC plots were calculated in each sample in FCS Express. For calibration samples, cells were further incubated for 5 min at 37°C in the presence of 10 µM valinomycin and 10 µM nigericin dissolved into cellular calibration pH buffers according to the instruction of the manufacturer, which was followed by measurement as above. Corresponding pH values were interpolated from the calibration curve.

### 2.6 Examination of time-dependent effects of ClGBI on the lysosomal pH of polarized macrophages

THP-1 cells seeded into 8-well chambered coverglass were differentiated into macrophages and polarized as above, and treated subsequently for 1, 4 or 18 h with 50 or 100 µM ClGBI supplemented into the culture media of cells. 3 hours before the measurement time, 70,000 MW, anionic dextrane conjugated to pH-sensitive fluorescein and pH-insensitive tetramethylrhodamine (TAMRA) (Thermo Fisher) was added to cells for 1 h at 37°C, which was followed by washing and a chase of 2 h at 37°C to ensure the exclusive lysosomal accumulation of the indicator (72). Live cell imaging was carried out in medium buffered with 20 mM HEPES, pH 7.4, and images were taken at the midplane of cells using an LSM880 confocal laser-scanning microscope (Carl Zeiss AG, Jena, Germany). Fluorescein and TAMRA were excited at 488 nm and 543 nm, respectively, and their emission was detected in the wavelength ranges of 493-559 nm and 559-685 nm, respectively. During image analysis in Matlab (Mathworks, Natick, MA), after manual selection of pixels corresponding to an individual cell based on the transmission image, a threshold value of the TAMRA fluorescence intensity was determined and pixels having larger TAMRA intensity than the threshold were identified as lysosomal pixels. Subsequently, the average fluorescein/TAMRA fluorescence intensity ratio positively correlating with the value of lysosomal pH was calculated for each cell using data of lysosomal pixels exclusively. For calibration samples, cells were incubated for 15 min at 37°C in the presence of 10 µM valinomycin, 10 µM nigericin and 0.1 µM bafilomycin dissolved into cellular calibration pH buffers according to the instruction of the manufacturer, which was followed by measurement as above. Corresponding pH values for each ratio were interpolated from the calibration curve.

### 2.7 Analysis of time-dependent effects of ClGBI on the plasma membrane ceramide levels of polarized macrophages

THP-1 cells seeded into 24-well plates were differentiated into macrophages and polarized as above, and treated subsequently for 1, 4 or 18 h with 50 or 100 µM ClGBI supplemented into the culture media of cells. After washing, detachment with accutase and FcR blocking as above, treated and control M0, M1 and M2 cells were labeled with anti-ceramide antibodies clone 15B4 (Sigma-Aldrich) at 4 µg/ml for 60 min at room temperature, which was followed by washing and a 20-min staining with Alexa Fluor 647-conjugated goat anti-mouse IgM antibody (Thermo Fisher Scientific) at 4 µg/ml at room temperature. The fluorescence intensity of individual cells was measured with NovoCyte 3000RYB using excitation at 640 nm and a 660/20 emission band pass filter and average fluorescence intensities of at least 10,000 cells with a normal morphology gated on FSC-SSC plots were calculated in each sample in FCS Express. The low extent of nonspecific binding of the primary antibody was supported by data obtained with the isotope control antibody Mouse IgM Isotype Control (Thermo Fisher) applied at identical experimental conditions (**Supplementary Figure 5**).

### 2.8 Statistical analysis

Measured data are represented as mean ± SEM obtained from *n* biological replicates for flow cytometry or *n* individual cells from five independent experiments for confocal microscopy, as indicated in figure legends. In measurements carried out with a flow cytometer, at least 10,000 cells per sample were analyzed in each independent experiment. The p values were calculated by Tukey’s HSD test carried out after significant differences were obtained for between-group effects in ANOVA. Differences were considered significant when p < 0.05 (*p < 0.05, **p < 0.01, ***p < 0.001, ****p < 0.0001).

## 3 Results

### 3.1 Establishment and validation of a THP-1-derived polarized macrophage model

**Figure 1:**
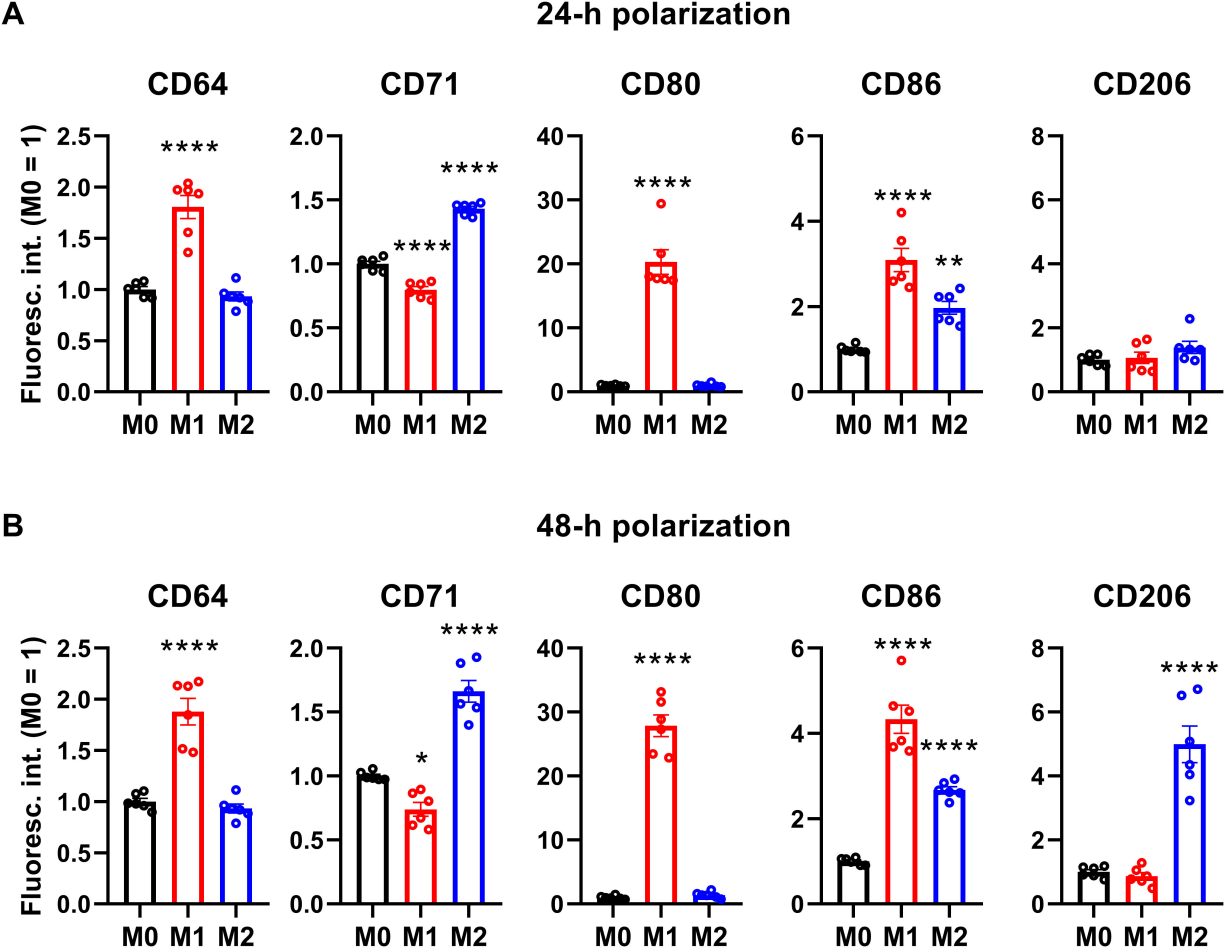
Cell surface CD marker expression profiles of M0, M1 and M2 THP-1-derived macrophages. THP-1 cells were differentiated into macrophages for 24 h by 10 ng/ml PMA, which was followed by a 24-h resting period in the absence of PMA. The differentiated M0 macrophages were subsequently polarized for 24 h **(A)** or 48 h **(B)** into classical M1 macrophages with 100 ng/ml LPS plus 20 ng/ml IFN-γ, or M2 macrophages using 20 ng/ml IL-4 plus 20 ng/ml IL-13. After accutase-mediated detachment and Fc receptor blocking, the cell surface expression of CD markers of differentiated and polarized macrophages was quantified using fluorophore-conjugated antibodies and flow cytometry. The average fluorescence intensity of at least 10,000 individual cells of normal morphology per sample was determined and subsequently normalized to the mean value of M0 samples. The normalized fluorescence intensity values obtained in n = 6 biological replicates, and their average values (± SEM) are plotted in both panels. Asterisks indicate significant differences compared to M0 samples (*p < 0.05, **p < 0.01, ****p < 0.0001, ANOVA followed by Tukey’s HSD test).

In order to generate a simple, efficient and reproducible experimental model of human polarized macrophages, we differentiated acute monocytic leukemia-derived THP-1 cells into macrophages using a low, 10 ng/ml concentration of PMA for 24 h, which, similar to what we observed in our previous studies (73, 74), resulted in attachment of the cells to the bottom of the culture dish, and substantial changes in cellular morphology typical for macrophages with reduced nucleocytoplasmic ratio. After resting PMA-differentiated macrophages for additional 24 h in medium lacking PMA or other activators to ensure M0 phenotype, the cells were polarized according to the classical M1 activation route via LPS and IFN-γ, or on the alternative M2 pathway using IL-4 and IL-13 (64–67). To validate our experimental model, the success of polarization was subsequently tested after 24 and 48 h of polarization by examining the cell surface expression pattern of CD markers characteristic for the different macrophages using flow cytometry. In agreement with literature data, THP-1-derived macrophages treated with LPS and IFN-γ for 24 or 48 h showed distinctive properties of M1 activation manifested in significantly elevated levels of CD64, CD80 and CD86, which were accompanied by no changes in CD206 expression and reduced CD71 levels (**Figure 1**; **Supplementary Figure 1**). In contrast, cells treated with IL-4 and IL-13 for 24 or 48 h displayed an M2-like CD marker expression profile with significantly increased amounts of cell surface CD71 and CD86 in the absence of changes in CD64 or CD80 expression. In addition, significantly higher expression of the M2-specific CD206 was found in IL-4 and IL-13-treated cells, however, that appeared only after 48 h. Furthermore, CD expression patterns were strongly similar after 24-h and 48-h polarization, which altogether imply that our generated M1 and M2 cells exhibit cell surface CD expression profiles characteristic for classically and alternatively activated macrophages (75, 76), respectively, already after 24 h polarization, which are retained at least for 48 h. Therefore, experiments described in the next sections and performed between 24 and 48 h of polarization were carried out in stably polarized macrophages.

### 3.2 The H_V_1 inhibitor ClGBI dose- and polarization-dependently reduces the viability of polarized macrophages

**Figure 2:**
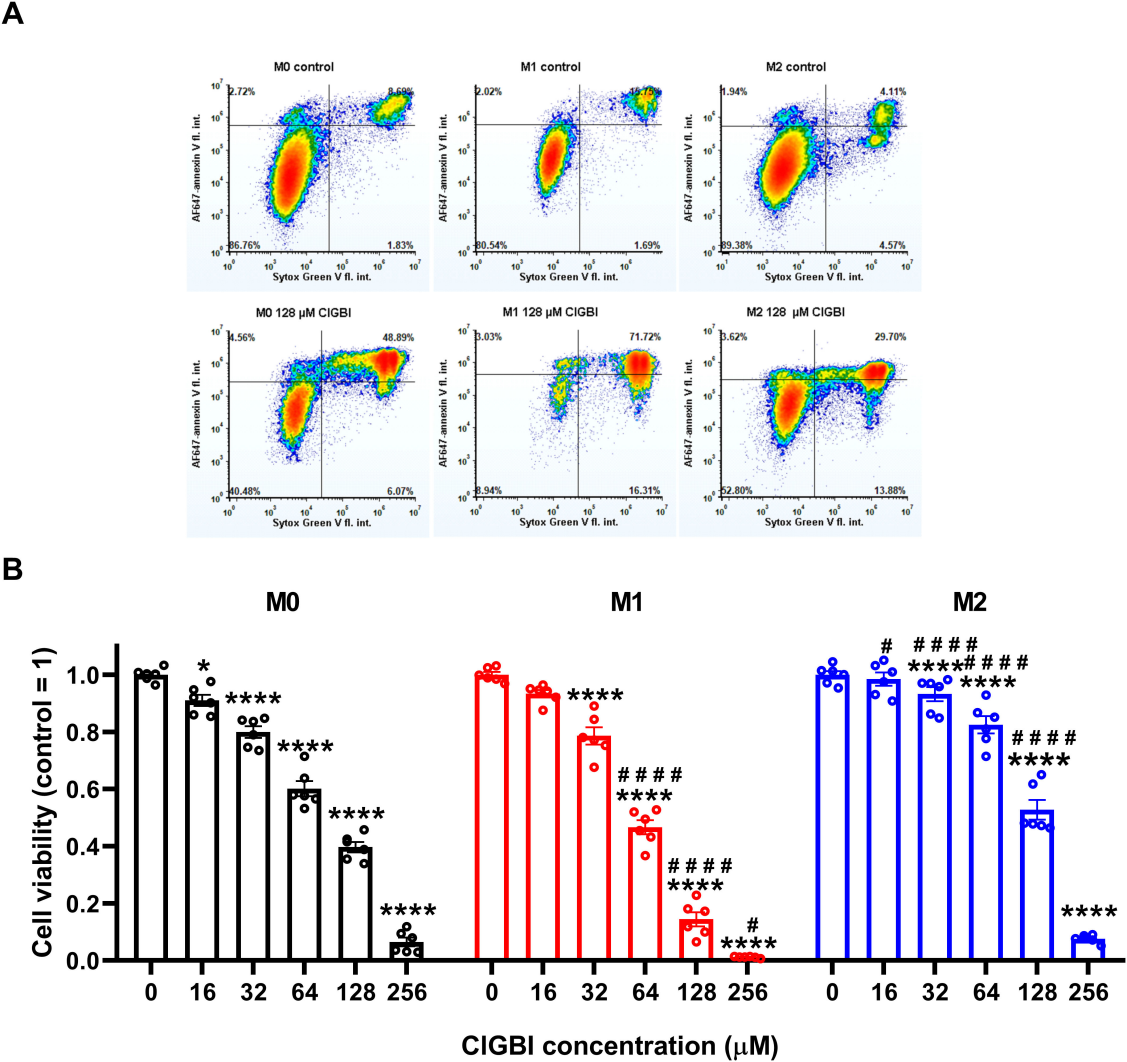
The H_V_1 inhibitor ClGBI dose-dependently compromises the viability of polarized macrophages most effectively in M1 cells. THP-1 cells were differentiated into macrophages for 24 h by 10 ng/ml PMA, which was followed by a 24-h resting period in the absence of PMA. The differentiated M0 macrophages were subsequently polarized for 24 h into classical M1 macrophages with 100 ng/ml LPS plus 20 ng/ml IFN-γ, or M2 macrophages using 20 ng/ml IL-4 plus 20 ng/ml IL-13. Cells were subsequently treated for 24 h with ClGBI at concentrations ranging between 16 and 256 µM. After collecting cells in suspension and those detached by accutase, the macrophages were labeled with Sytox Green and Alexa Fluor 647-conjugated annexin V to identify necrotic and apoptotic cells, respectively. Fluorescence intensities of individual cells were measured using flow cytometry and the relative fraction of double negative viable cells was determined in each sample containing at least 10,000 cells, and normalized to the mean value determined in control untreated samples. **(A)** Representative density plots demonstrate the effect of 128 µM ClGBI on the viability of M0, M1 and M2 macrophages. **(B)** The normalized viable ratios obtained in n = 6 biological replicates, and their average values (± SEM) are plotted in the figure. Asterisks indicate significant differences compared to control samples (*p < 0.05, ****p < 0.0001), while hashes show that compared to M0 at the given applied ClGBI concentration (^#^p < 0.05, ^####^p < 0.0001), which were determined by Tukey’s HSD test carried out after significant differences were obtained for between-group effects in ANOVA.

To test whether H_V_1 inhibition compromises the viability of polarized macrophages similarly to that described in various cell types (9, 14, 20, 23, 25, 38), we treated THP-1-derived macrophages polarized as above with a concentration series of ClGBI, a widely applied inhibitor of the channel (8, 9, 13, 14, 20–25). For this, ClGBI-treated and control cells were gently detached with accutase, which was previously shown not to affect cell viability (68), and subsequently labeled with Sytox Green and Alexa Fluor 647-conjugated annexin V to identify necrotic and apoptotic cells, respectively, and determine the fraction of double negative viable cells using flow cytometry (53, 69, 70). In all three examined THP-1-derived macrophage populations, ClGBI significantly and dose-dependently reduced the fraction of living cells (**Figure 2**). Notably, ClGBI resulted in dramatically lower cell viability at 128 and 256 µM, i.e. concentrations widely applied in cellular studies examining the functions of H_V_1 (8, 13, 14, 21, 22). Furthermore, the sensitivity of cells varied among the different macrophages as, at most examined concentrations, the effect induced in M1 cells was significantly higher, while that of M2 macrophages significantly lower than in the case of resting M0 macrophages.

### 3.3 The ClGBI-induced decrease in viability of polarized macrophages is mediated through H_V_1 inhibition

**Figure 3:**
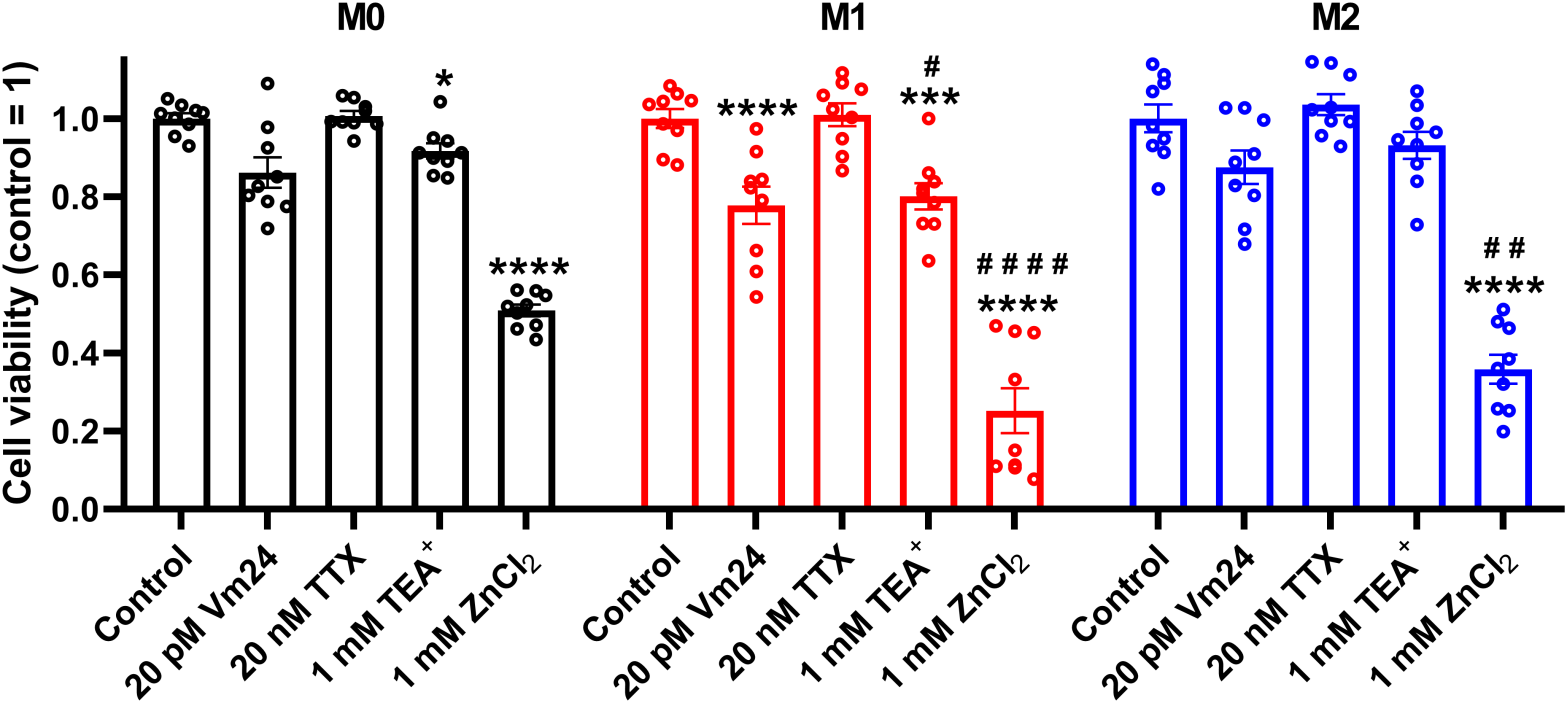
Effects of ion channel blockers on the viability of polarized macrophages. THP-1 cells were differentiated into macrophages for 24 h by 10 ng/ml PMA, which was followed by a 24-h resting period in the absence of PMA. The differentiated M0 macrophages were subsequently polarized for 24 h into classical M1 macrophages with 100 ng/ml LPS plus 20 ng/ml IFN-γ, or M2 macrophages using 20 ng/ml IL-4 plus 20 ng/ml IL-13. Cells were subsequently treated for 24 h with 20 pM Vm24 scorpion toxin, 20 nM TTX, 1 mM TEA^+^ or 1 mM (nominal) ZnCl_2_. After collecting cells in suspension and those detached by accutase, the macrophages were labeled with Sytox Green and Alexa Fluor 647-conjugated annexin V to identify necrotic and apoptotic cells, respectively. Fluorescence intensities of individual cells were measured using flow cytometry and the relative fraction of double negative viable cells was determined in each sample containing at least 10,000 cells, and normalized to the mean value determined in control untreated samples. The normalized viable ratios obtained in n = 9 biological replicates, and their average values (± SEM) are plotted in the figure. Asterisks indicate significant differences compared to control samples (*p < 0.05, ***p < 0.001, ****p < 0.0001), while hashes show that compared to M0 at the given applied inhibitor concentration (^#^p < 0.05, ^##^p < 0.01, ^####^p < 0.0001), which were determined by Tukey’s HSD test carried out after significant differences were obtained for between-group effects in ANOVA.

To corroborate that ClGBI-induced reductions in cell viability are induced by H_V_1 inhibition and not the off-target effects of the compound on other macrophage-resident ion channels (77), we examined the impact of other relevant ion channel blockers on the viability of M0, M1 and M2 THP-1-derived macrophages by applying Vm24 scorpion toxin, a specific blocker of K_V_1.3 (78), tetrodotoxin, a specific inhibitor of voltage-gated sodium channels (79), TEA^+^, a wide-spectrum potassium channel blocker (80), and Zn^2+^, a well-known non-specific inhibitor of H_V_1 (1). Vm24, tetrodotoxin, TEA^+^ and Zn^2+^ were applied at concentrations most commonly utilized in *in vitro* functional assays and cellular studies. In these experiments, TTX failed to affect cell viability, and the potassium channel inhibitors Vm24 or TEA^+^ resulted in only mild effects mainly in M1 cells. On the contrary, the H_V_1 inhibitor Zn^2+^ remarkably reduced the viability of macrophages, particularly in M1 cells in accordance with ClGBI effects (**Figure 3**). While the effect induced by Zn^2+^ on M1 macrophages was significantly larger than that on M0 cells, the difference between M1 and M2 was on the verge of statistical significance (p=0.0678). Although the significant difference between the effect of ClGBI on the viability of M1 and M2 macrophages would suggest a similar correlation for the effect of Zn^2+^, the lack of a smaller effect of Zn^2+^ on the viability of M2 macrophages can potentially be attributed to the importance of Zn-sensitive proteins in the function of M2 macrophages and consequent potential off-target effects or the lower potency of Zn^2+^ compared to ClGBI (81).

**Figure 4:**
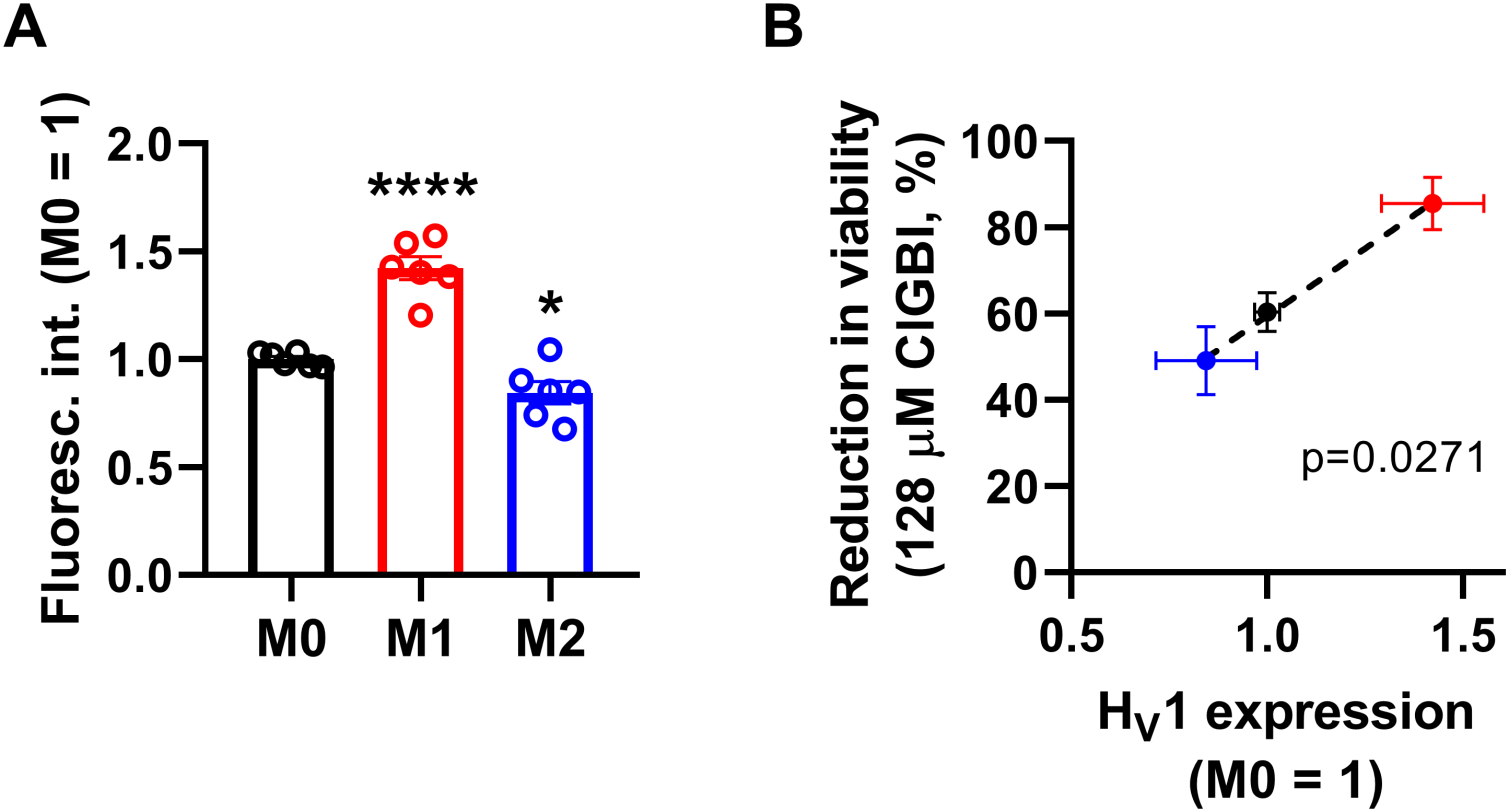
The H_V_1 expression of polarized macrophages correlates with the ClGBI-induced reduction in cell viability. **(A)** THP-1 cells were differentiated into macrophages for 24 h by 10 ng/ml PMA, which was followed by a 24-h resting period in the absence of PMA. The differentiated M0 macrophages were subsequently polarized for 24 h into classical M1 macrophages with 100 ng/ml LPS plus 20 ng/ml IFN-γ, or M2 macrophages using 20 ng/ml IL-4 plus 20 ng/ml IL-13. After accutase-mediated detachment, fixation, permeabilization and Fc receptor blocking, the H_V_1 expression of differentiated and polarized macrophages was quantified using indirect immunofluorescence labeling and flow cytometry. The average fluorescence intensity of at least 10,000 individual cells of normal morphology per sample was determined and subsequently normalized to the mean value of M0 samples. The normalized fluorescence intensity values obtained in n = 6 biological replicates, and their average values (± SEM) are plotted in the figure. Asterisks indicate significant differences compared to M0 samples (*p < 0.05, ****p < 0.0001, ANOVA followed by Tukey’s HSD test). **(B)** Average values (± SEM) of reduction in the viability of M0, M1 and M2 macrophages induced by 128 µM ClGBI as a function of H_V_1 expression normalized to M0 cells. The *p* value determined with Deming regression analysis is shown in the panel, which revealed significant correlation between the extent of compromised cell viability and H_V_1 expression.

Since both of the examined H_V_1 blockers, ClGBI and Zn^2+^, compromised the viability of M1 macrophages to a larger extent than in M0 or M2 cells (**Figures 2**, **3**), we next investigated the H_V_1 expression in these cells using flow cytometry. Consistent with the larger effects of H_V_1 blockers, M1 cells exhibited significantly higher H_V_1 expression than M0 cells, while the abundance of H_V_1 was the lowest in M2 cells (**Figure 4A**; **Supplementary Figure 2**). Furthermore, an excellent correlation was found between the extents of reductions in the viability of M0, M1 and M2 macrophages and the levels of H_V_1 expression (**Figure 4B**). Altogether, the results obtained with other relevant ion channel inhibitors and the correlation between H_V_1 expression and the extent of H_V_1 inhibition-induced reductions in cell viability convincingly support that the effects elicited by ClGBI may in fact be caused by its inhibitory actions on H_V_1.

### 3.4 ClGBI-induced alterations in cytoplasmic and lysosomal pH of polarized macrophages

**Figure 5:**
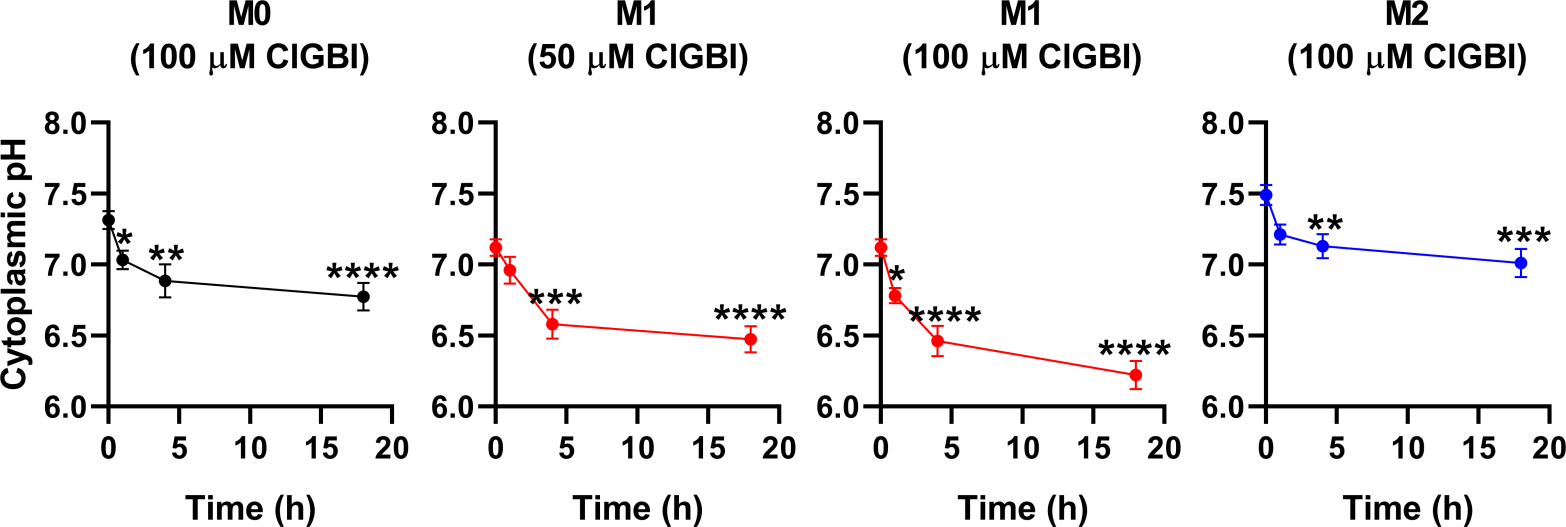
ClGBI time-dependently acidifies the cytoplasm of polarized macrophages most efficiently in M1 cells. THP-1 cells were differentiated into macrophages for 24 h by 10 ng/ml PMA, which was followed by a 24-h resting period in the absence of PMA. The differentiated M0 macrophages were subsequently polarized for 24 h into classical M1 macrophages with 100 ng/ml LPS plus 20 ng/ml IFN-γ, or M2 macrophages using 20 ng/ml IL-4 plus 20 ng/ml IL-13. Cells were subsequently treated for 1, 4 or 18 h with 50 or 100 µM ClGBI. In the last 30 min of incubation, the cytoplasmic pH indicator pHrodo Red AM was further added to cells. After accutase-mediated detachment, the fluorescence intensity of individual cells was measured using flow cytometry and the average fluorescence intensity of at least 10,000 cells of normal morphology per sample was determined. Corresponding pH values of individual cells were subsequently interpolated from the calibration curve determined based on calibration samples incubated with valinomycin and nigericin dissolved into cellular calibration pH buffers. The average pH values obtained from n = 12 biological replicates (± SEM) are plotted in the figure. Asterisks indicate significant differences compared to untreated control samples (*p < 0.05, **p < 0.01, ***p < 0.001, ****p < 0.0001, ANOVA followed by Tukey’s HSD test).

Since an H_V_1 deficiency or inhibition was previously shown to affect pH regulation by inducing cytoplasmic acidification in different cell types (8, 10, 13, 18, 20, 23, 28, 36, 82), we next investigated changes in the pH of the cytoplasm of polarized macrophages by using flow cytometry and pHrodo Red AM, an intracellular pH-sensitive fluorescence indicator that traverses the cell membrane and remains within the intracellular space upon cleavage by nonspecific esterases (71). In these measurements, we applied ClGBI for various durations at concentrations roughly corresponding to the dose eliciting 50% decreases in cell viability, i.e. 100 µM for M0 and M2 cells and 50 µM for M1 cells. Furthermore, to ensure comparability of sensitivities, we carried out experiments by adding 100 µM to M1 cells as well. Given that cell death is generally associated with losing adherence, in these experiments and those described later when examining the mechanism of ClGBI action, we analyzed only the cells that remained adherent to the cell culture dish after the treatment to investigate molecular events leading to cell death and not solely appearing as a consequence of it. We observed time-dependent increases in the fluorescence intensity of the dye in all macrophage populations referring to a pH reduction. To quantify these alterations, we performed calibration with samples incubated in the presence of a combination of valinomycin and nigericin dissolved into cellular calibration buffers of known pH. When interpolating pH values of samples from the calibration curve, we found significant time-dependent pH decreases in all macrophage subtypes in response to ClGBI (**Figure 5**; **Supplementary Figure 3**). Notably, the magnitudes of changes were the largest in M1 cells, particularly at 100 µM concentration of the compound, in accordance with the higher H_V_1 expression of these cells.

**Figure 6:**
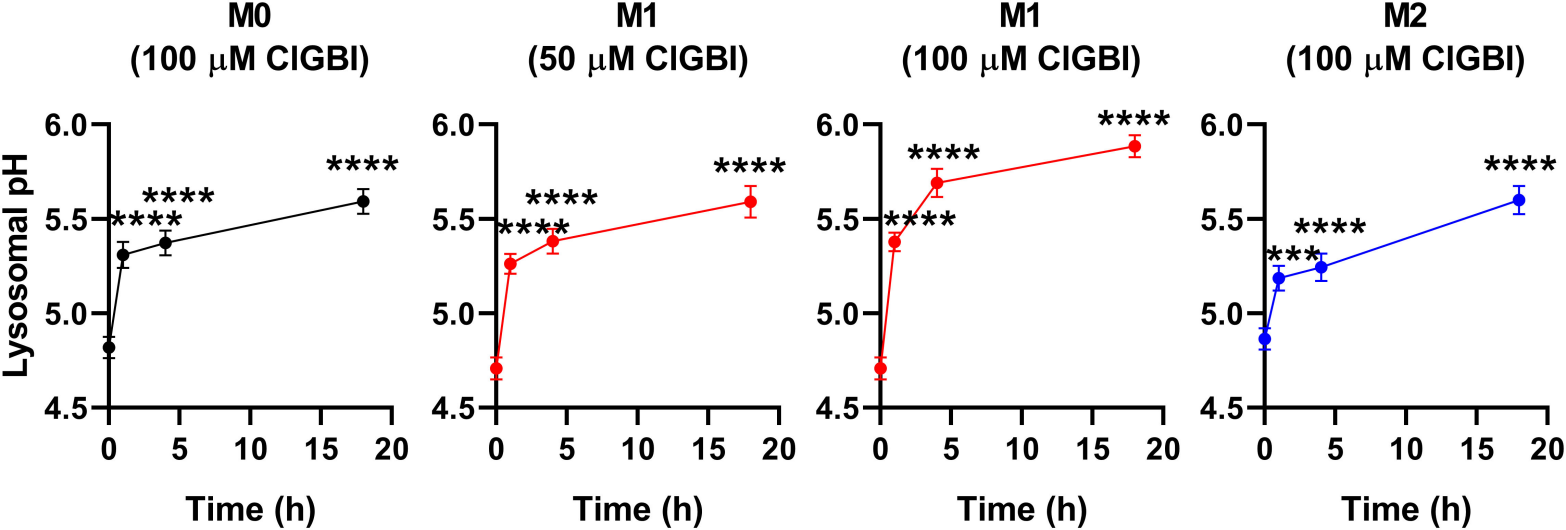
ClGBI time-dependently alkalizes the lyosomes of polarized macrophages most efficiently in M1 cells. THP-1 cells were differentiated into macrophages for 24 h by 10 ng/ml PMA, which was followed by a 24-h resting period in the absence of PMA. The differentiated M0 macrophages were subsequently polarized for 24 h into classical M1 macrophages with 100 ng/ml LPS plus 20 ng/ml IFN-γ, or M2 macrophages using 20 ng/ml IL-4 plus 20 ng/ml IL-13. Cells were subsequently treated for 1, 4 or 18 h with 50 or 100 µM ClGBI. 3 hours before the measurement time, 70,000 MW, anionic dextrane conjugated to pH-sensitive fluorescein and pH-insensitive tetramethylrhodamine (TAMRA) was added to cells for 1 h, which was followed by a chase of 2 h. Then, images were taken at the midplane of cells using a confocal microscope and the average fluorescein/TAMRA fluorescence intensity ratio positively correlating with the value of lysosomal pH was calculated for each individual cell using data of pixels corresponding to lysosomes. pH values were subsequently interpolated from the calibration curve determined based on calibration samples incubated with valinomycin, nigericin and bafilomycin dissolved into cellular calibration pH buffers. The average pH values obtained from n = 60-80 individual cells obtained from five independent experiments (± SEM) are plotted in the figure. Asterisks indicate significant differences compared to untreated control samples (***p < 0.001, ****p < 0.0001, ANOVA followed by Tukey’s HSD test).

While lysosomal pH alterations in response to H_V_1 inhibition and their potential link to compromised viability have not been examined previously in any cell types, based on the facts that a deficiency or a block of the channel modifies phagosomal pH acidification (49, 50) and reduced lysosomal acidity is often associated with cell death (82, 83), it is reasonable to assume that such lysosomal pH changes may occur due to reduced H_V_1 conductance, which could contribute to cellular toxicity. To confirm this hypothesis, we evaluated ClGBI-induced effects on lysosomal pH. For this, polarized macrophages were treated with ClGBI as described above, and also incubated for 1 h in the presence of 70,000 MW, anionic dextrane conjugated to a pH-sensitive and a pH-insensitive fluorophore, fluorescein and tetramethylrhodamine, respectively, which was followed by a chase period in the absence of the dextrane for 2 h. This protocol ensured that the indicator is exclusively accumulated into the lysosomes (72). Hence, fluorescence microscopic determination of the Fluorescein/TAMRA ratio quantifies the lysosomal pH of cells. Fluorescein/TAMRA ratios can be directly translated into lysosomal pH values by applying calibration samples with valinomycin, nigericin and bafilomycin dissolved into cellular calibration buffers of known pH. By using this method, we observed significant time-dependent alkalinization of the lysosomes of M0, M1 and M2 macrophages as well in response to blocking H_V_1 channels with ClGBI (**Figure 6**; **Supplementary Figure 4**). Again, the largest effects were found in M1 macrophages at 100 µM ClGBI. Altogether, our results suggest a profound pH dysregulation throughout the cell in response to H_V_1 inhibition, which involves both the cytoplasmic and lysosomal compartments.

### 3.5 ClGBI-induced reductions in cell viability are associated with parallel time-dependent elevations of plasma membrane ceramide levels

**Figure 7:**
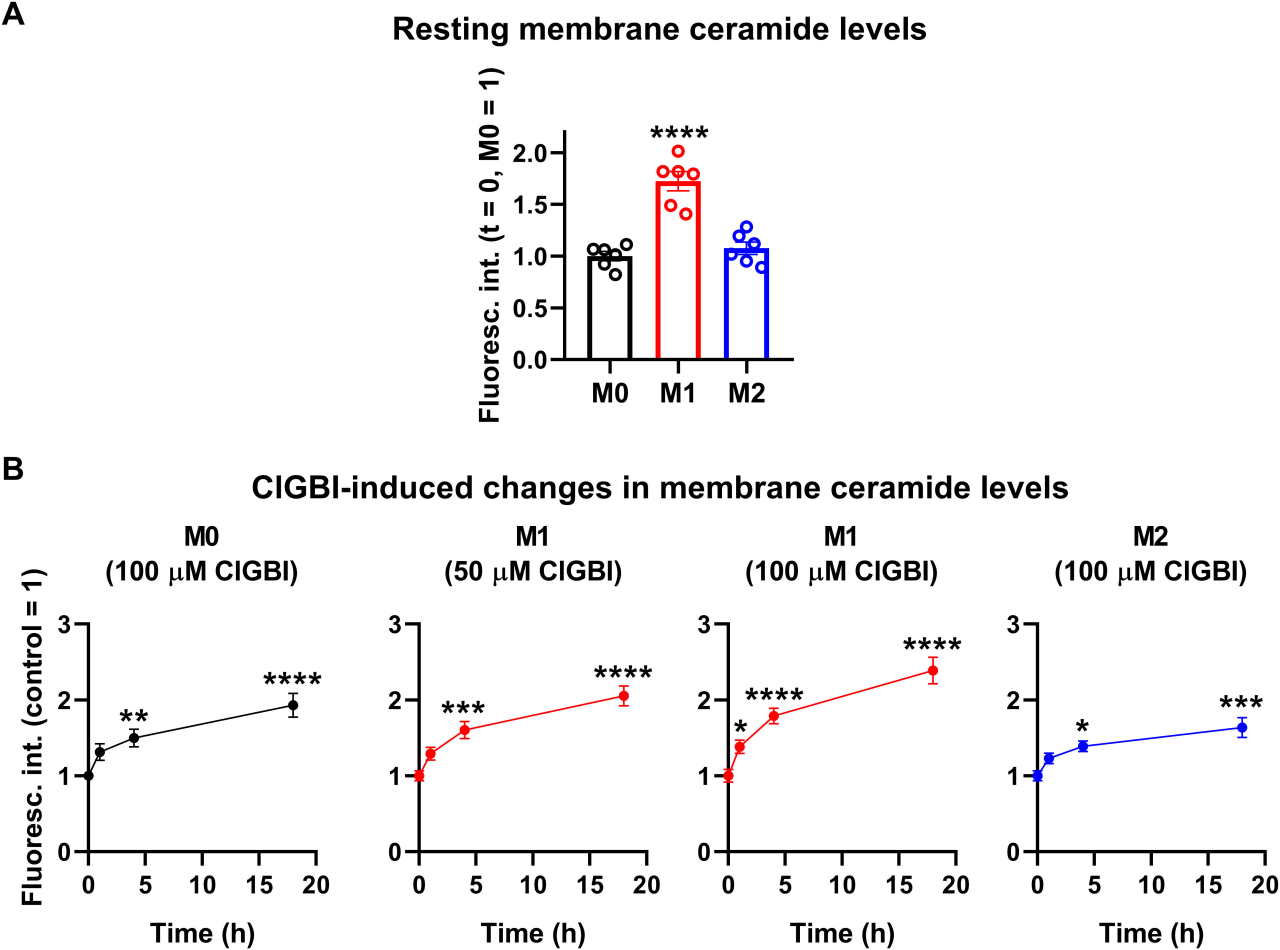
ClGBI induces a time-dependent elevation in membrane ceramide levels of polarized macrophages most effectively in M1 cells. THP-1 cells were differentiated into macrophages for 24 h by 10 ng/ml PMA, which was followed by a 24-h resting period in the absence of PMA. The differentiated M0 macrophages were subsequently polarized for 24 h into classical M1 macrophages with 100 ng/ml LPS plus 20 ng/ml IFN-γ, or M2 macrophages using 20 ng/ml IL-4 plus 20 ng/ml IL-13. Cells were subsequently treated for 1, 4 or 18 h with 50 or 100 µM ClGBI. After accutase-mediated detachment and Fc receptor blocking, cells were labeled with anti-ceramide antibodies followed by AlexaFluor647-conjugated goat anti-mouse IgM antibodies. Fluorescence intensities of individual cells were subsequently measured using flow cytometry and the average fluorescence intensity of at least 10,000 cells of normal morphology per sample was determined. **(A)** Average fluorescence intensities of each untreated sample were subsequently normalized to the mean value determined in the untreated M0 macrophages and the normalized fluorescence intensity values obtained in n = 6 biological replicates, and their average values (± SEM) are plotted in the panel. Asterisks indicate significant differences compared to M0 samples (****p < 0.0001, ANOVA followed by Tukey’s HSD test). **(B)** Alternatively, for each cell type, average fluorescence intensities of each sample were normalized to the mean value determined in the untreated, control sample. The average values (± SEM) of normalized fluorescence intensities obtained from n = 6 biological replicates are plotted in the figure. Asterisks indicate significant differences compared to untreated control samples (*p < 0.05, **p < 0.01, ***p < 0.001, ****p < 0.0001, ANOVA followed by Tukey’s HSD test).

While experiments outlined in the previous section demonstrated pH alterations, they did not provide information about the possible molecular mechanisms connecting the reduced H_V_1 conductance, complex pH dysregulation and cell death. Considering the substantial link between increased ceramide levels and various forms of cell death (55, 57) and the fact that ceramide is synthetized by pH-sensitive enzymes (58–60), we hypothesized that an elevation in membrane ceramide levels contributes to the effects of H_V_1 inhibitors on cell viability. To confirm this assumption, first we quantified time-dependent changes in the membrane abundance of ceramide using flow cytometry and anti-ceramide antibodies as previously (53). First, we compared membrane ceramide levels of untreated samples and found that the membrane abundance of ceramide was significantly higher in M1 cells than in M0 or M2 macrophages (**Figure 7A**; **Supplementary Figure 5**). Furthermore, in these measurements, ClGBI resulted in significant time-dependent increases in membrane ceramide levels in M0, M1 and M2 macrophages as well, and the largest elevation was seen in M1 cells in response to 100 µM ClGBI (**Figure 7B**; **Supplementary Figure 5**). These experiments convincingly showed that a H_V_1 inhibition was accompanied by increased ceramide levels, however, they did not provide any details about the exact molecules involved in the plausible causative relationship between the two.

### 3.6 ClGBI-induced reductions in cell viability are alleviated by an inhibitor of acid sphingomyelinase

**Figure 8:**
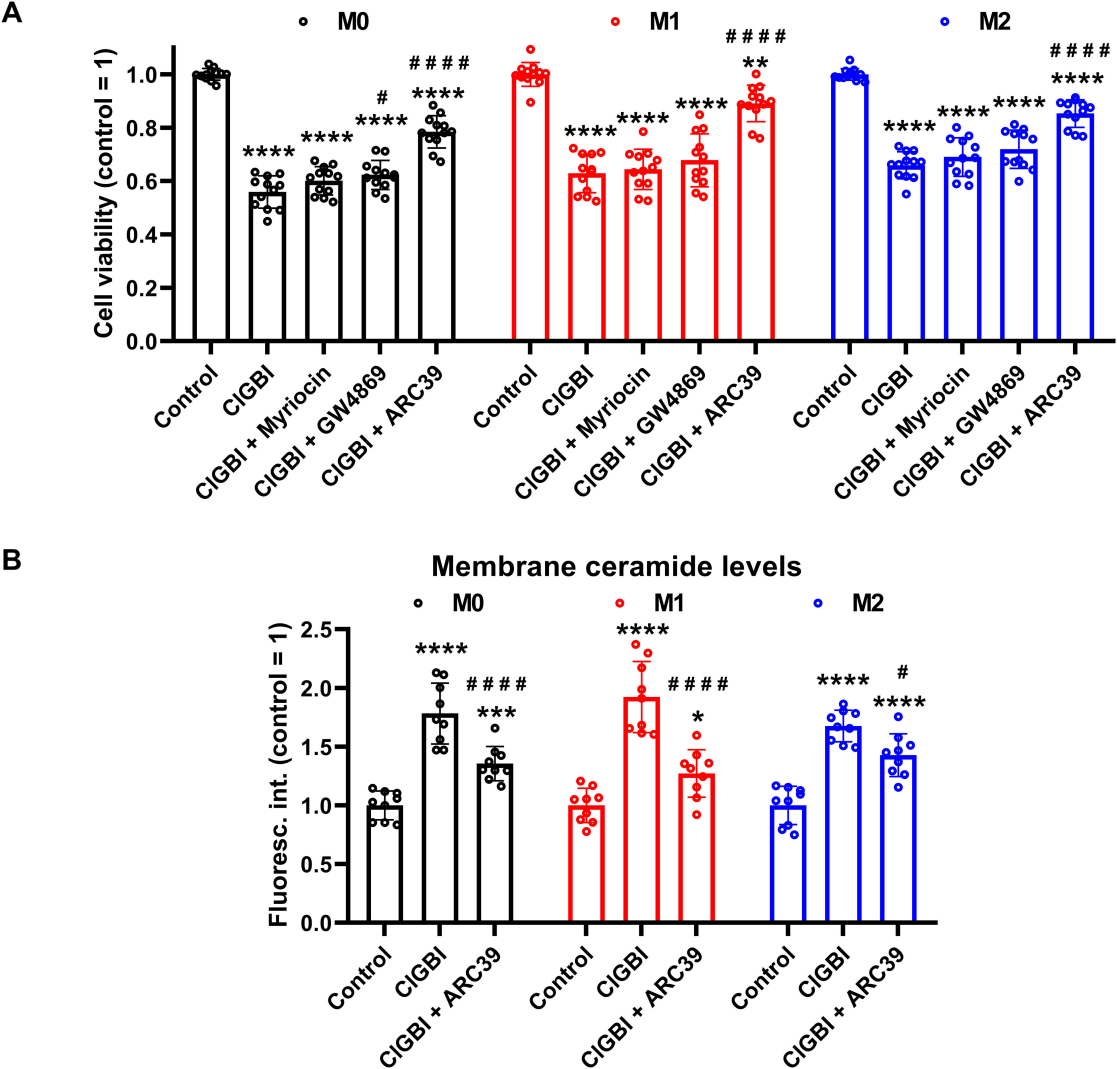
Protective effects of ceramide production inhibitors against the ClGBI-induced toxicity in polarized macrophages. **(A)** THP-1 cells were differentiated into macrophages for 24 h by 10 ng/ml PMA, which was followed by a 24-h resting period in the absence of PMA. The differentiated M0 macrophages were subsequently polarized for 24 h into classical M1 macrophages with 100 ng/ml LPS plus 20 ng/ml IFN-γ, or M2 macrophages using 20 ng/ml IL-4 plus 20 ng/ml IL-13. Cells were then pre-treated with 1 µM of the serine palmitoyltransferase inhibitor myriocin, 2 µM of the neutral sphingomyelinase blocker GW4869 or 5 µM of the acidic sphingomyelinase inhibitor ARC39 for 30 min, which was followed by a 24-h application of 100 µM ClGBI in M0 and M2 cells, or 50 µM ClGBI in M1 macrophages. After collecting cells in suspension and those detached by accutase, the macrophages were labeled with Sytox Green and Alexa Fluor 647-conjugated annexin V to identify necrotic and apoptotic cells, respectively. Fluorescence intensities of individual cells were measured using flow cytometry and the relative fraction of double negative viable cells was determined in each sample containing at least 10,000 cells, and normalized to the mean value determined in control untreated samples. The normalized live fractions obtained in n = 12 biological replicates, and their average values (± SEM) are plotted in the figure. Asterisks indicate significant differences compared to untreated, control samples (**p < 0.01, ****p < 0.0001), while hashes show that compared to cells treated only with ClGBI (^#^p < 0.05, ^####^p < 0.0001), which were determined by Tukey’s HSD test carried out after significant differences were obtained for between-group effects in ANOVA. **(B)** THP-1 cells differentiated, rested and polarized as above were pre-treated with 5 µM ARC39 for 30 min, which was followed by a 24-h application of 100 µM ClGBI in M0 and M2 cells, or 50 µM ClGBI in M1 macrophages. After accutase-mediated detachment and Fc receptor blocking, cells were labeled with anti-ceramide antibodies followed by AlexaFluor647-conjugated goat anti-mouse IgM antibodies. Fluorescence intensities of individual cells were subsequently measured using flow cytometry and the average fluorescence intensity of at least 10,000 cells of normal morphology per sample was determined. For each cell type, average fluorescence intensities of each sample were normalized to the mean value determined in the untreated, control sample. The normalized fluorescence intensity values obtained in n = 9 biological replicates, and their average values (± SEM) are plotted in the panel. Asterisks indicate significant differences compared to untreated, control samples (*p < 0.05, ***p < 0.001, ****p < 0.0001), while hashes show that compared to cells treated only with ClGBI (^#^p < 0.05, ^####^p < 0.0001), which were determined by Tukey’s HSD test carried out after significant differences were obtained for between-group effects in ANOVA.

If elevated membrane ceramide levels indeed contributed to the compromised cell viability induced by an H_V_1 block, such an effect should be alleviated by an inhibition of ceramide synthesis. Since ceramide production mainly occurs through *de novo* synthesis, or activation of neutral and acid sphingomyelinases (55–57), we examined the potential protective effects of inhibitors of these pathways. Namely, we inhibited serine palmitoyltransferase with myriocin (84), neutral sphingomyelinase with GW4869 (85), and acid sphingomyelinase with ARC39 (86). In these experiments THP-1-derived macrophages were pre-treated with these inhibitors, which was followed by an incubation in the presence of ClGBI, 100 µM in the case of M0 and M2 cells while 50 µM in M1 macrophages, and determination of the fraction of viable cells as above (**Figure 2**). In accordance with our previous experiments, ClGBI largely reduced viability in M0, M1 and M2 cells as well (**Figure 8A**). However, inhibitors of ceramide production provided partial protection against ClGBI. While such effects induced by the serine palmitoyltransferase inhibitor myriocin and the neutral sphingomyelinase blocker GW4869 were rather negligible, ARC39, that specifically inhibits acid sphingomyelinase, largely alleviated ClGBI-induced compromised viabilities in all cell types, but particularly in M1 macrophages. Furthermore, ClGBI-elicited increases in plasma membrane ceramide levels were attenuated by ARC39 in all macrophage subtypes (**Figure 8B**). Altogether, these results confirmed that blocking H_V_1 results in cellular toxicity, which is mediated by an overproduction of ceramide, mainly through acid sphingomyelinase activity.

## 4 Discussion

**Figure 9:**
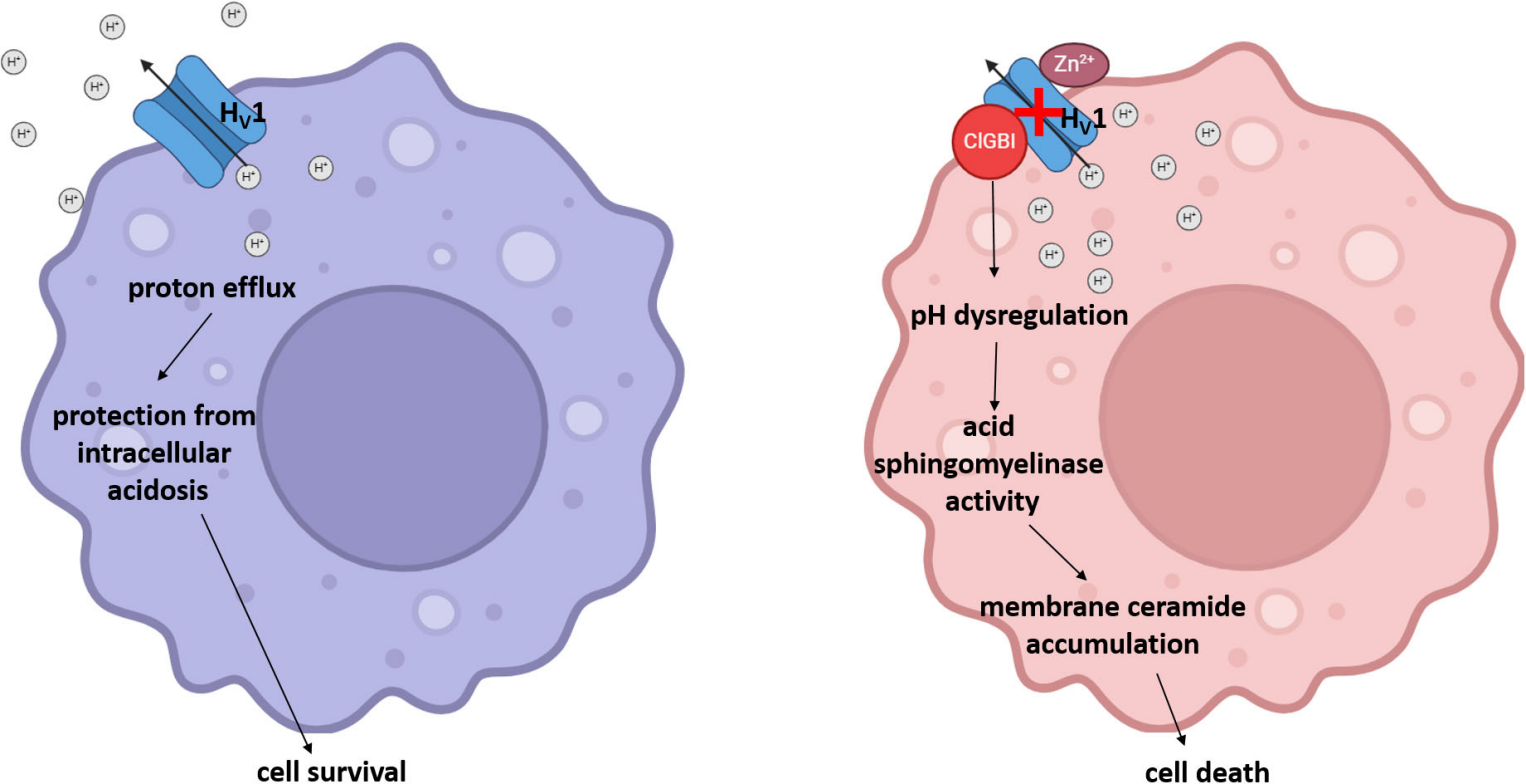
The potential molecular mechanism of the H_V_1 inhibition-induced compromised viability of polarized macrophages. Under normal circumstances, the H_V_1-mediated proton extrusion from the cytoplasm protects cells from intracellular acidosis and consequent cell death. However, a pharmacological H_V_1 inhibition leads to a complex pH dysregulation throughout the cell involving cytoplasmic and lysosomal compartments, which results in ceramide overproduction mainly through the activity of acid sphingomyelinase in polarized macrophages, and eventually the compromised viability of cells.

Considering that H_V_1 inhibitors show great therapeutic promise in various human pathological conditions such as neuroinflammation and malignant diseases, understanding the effects of channel blockers exerted on the viability of macrophages with different polarization is of crucial importance regarding both the therapeutic effects and the potential side effects of these blockers. This study highlights that ClGBI, the most widely used inhibitor of H_V_1, compromises the viability of THP-1-derived polarized macrophages at concentrations typically applied in *in vitro* functional tests, and M1 macrophages show superior sensitivity to this compound when compared to M0 and, in particular, M2 cells. Our results indicate that ClGBI indeed elicits this action primarily by blocking H_V_1 since i) the ClGBI sensitivity of M0, M1 and M2 macrophages shows strong correlation with their H_V_1 expression levels, i.e. M1 macrophages with the highest H_V_1 abundance are the most susceptible; ii) inhibitors of other ion channels that are relevant in macrophage functions and potentially also blocked by ClGBI cause no notable reductions in viability; and iii) Zn^2+^, another non-specific H_V_1 blocker, shows a similar tendency to reduce cell viability as ClGBI. In our experiments, ClGBI concomitantly induced a complex cellular pH dysregulation manifested in acidification of the cytoplasm and alkalinization of the lysosomes, which eventually resulted in the accumulation of membrane ceramides. Highlighting the fundamental role of the ceramide level elevation in the process, the compromised cell viability in response to ClGBI in M0, M1 and M2 macrophages was effectively alleviated by a pre-treatment with ARC39, a specific inhibitor of the pH-sensitive acid sphingomyelinase. Altogether, our data suggest that H_V_1 inhibition leads to cellular toxicity in polarized macrophages in a polarization-dependent manner, which occurs due to a pH dysregulation and concomitant ceramide overproduction mainly depending on the activity of the pH-sensitive acid sphingomyelinase (**Figure 9**).

In our study, we utilized a model system of polarized M1 and M2 macrophages based on macrophages differentiated from human THP-1 moncytoid cells. THP-1-derived macrophages are frequently applied for a large variety of purposes in medical research (87, 88). In our experimental setup, we first differentiated THP-1 cells into macrophages using 10 ng/ml PMA, an activator of protein kinase C, just as in our previous studies (73, 74). Given that higher concentrations of PMA can result in an undesirable induction of gene expression during macrophage differentiation leading to an M1-like phenotype, we applied such a low PMA concentration to maintain the responsiveness of macrophages to secondary stimuli (61, 62). Moreover, after differentiation, the resulting macrophages were rested for 24 h in medium lacking PMA to ensure their M0 phenotype more closely resembling that of primary human monocyte-derived macrophages and retain their plasticity to stimulus-directed polarization (61, 63). The differentiated M0 macrophages were subsequently polarized into classical M1 macrophages with LPS and IFN-γ, or M2 macrophages using IL-4 and IL-13. While concerns were raised previously regarding translational limitations of results obtained with THP-1-derived macrophages mainly in cases of alternative polarization pathways (89–91), THP-1-derived macrophages remain widely applied and generally accepted as simplified, suitable and reliable biologically relevant model systems. This is justified by their easy and safe use, storage and recovery, purity, good polarizability, homogenous genetic background minimizing the degree of variability in the cell phenotype, their human origin that avoids problems regarding the translatability of results obtained with mouse models resulting from major differences between the human and mouse immune system, and their ability to mimic functional activities of both M1 and M2 macrophages after an appropriate polarization (87, 88). Accordingly, our treatments, similarly to those described by other studies applying similar polarization protocols in THP-1-derived macrophages as well (64–67), resulted in CD expression profiles characteristic of M1 and M2 macrophages (75, 76), respectively. In particular, LPS plus IFN-γ-induced polarization caused upregulation of M1 markers such as CD64, CD80 and CD86, while IL-4 and IL-13 treated cells exhibited higher levels of M2 markers such as CD71 and CD86 (**Figure 1**). Furthermore, the latter were characterized by increased abundance of CD206, a traditional M2 marker, however, only at later time points. While the delayed appearance of CD206 might suggest inappropriateness of our M2 model at first glance, that could rather be attributed to the application accutase to detach cells for flow cytometry analysis. Namely, accutase was previously shown to artificially reduce levels of certain cell surface markers such as CD163 or CD206 while leaving the expression of antigen presentation markers such as CD80 or CD86, and FcγR markers such as CD64 unaffected (92), which could explain the lack of notable increases of CD206 in our experiments in M2 cells in the case of a 24-h polarization (**Figure 1**). In spite of this disadvantage, we utilized accutase to detach macrophages throughout our study, particularly in viability experiments, since it does not induce substantial changes in polarization and cell surface protein levels like trypsinization, and does not lead to reduced viability as EDTA-mediated detachment or cell scraping (68). While the expression of CD206 was found rather modest in our IL-4- and IL-13-polarized cells, considering its previously published delayed appearance in THP-1-derived M2 macrophages (65) and the potential reduction of its membrane expression due to accutase treatment, even such a moderate elevation demonstrates the success of our polarization protocol. Results of our CD marker analysis imply that polarized phenotypes are established already after 24 h and retained at least for 48 h, i.e. stable during the duration of our viability experiments. Altogether, while keeping in mind its potential limitations, our experimental model based on human THP-1-derived macrophages is appropriate to examine the functional importance of H_V_1 in polarized macrophages.

The fundamental roles of H_V_1 are widely documented in human macrophages. The presence of an effective proton-conducting extrusion pathway regulated by membrane potential and pH has long been described in mouse peritoneal macrophages, which was suggested to contribute to alkalinization of the cytoplasm thereby providing protection against intracellular acidification associated with activation and microbicidal mechanisms of these cells (93). Similar H_V_1 currents were also found in PMA-differentiated THP-1-derived non-polarized macrophages (46). In accordance with the suggested role of H_V_1 in the compensatory charge movement during the oxidative burst process, in mouse bone marrow-derived macrophages, a sustained ROS production induced by PMA and tumor necrosis factor-α (TNF-α) was reduced in response to its inhibition by Zn^2+^ (48), while the genetic deficiency of the channel resulted in delayed acidification and lower ROS production of phagosomes (50). Furthermore, H_V_1-silenced or H_V_1^-/-^ mouse bone marrow-derived macrophages showed attenuated ROS production and compromised protection from infection with the intracellular fungal pathogen *Histoplasma capsulatum* (51). Additionally, H_V_1 inhibition by Zn^2+^ modified phagosomal pH acidification in human peripheral blood monocyte-derived macrophages and the channel was suggested to contribute to the different phagosomal pH regulation and ROS production ability of macrophages polarized along the M1 and M2 pathways (49). While the expression of H_V_1 is relatively well-documented in macrophages, the effects of its inhibition on the viability of polarized cells have not been explicitly tested before in spite of the fact that blocking H_V_1 resulted in compromised viability in various cell types including Jurkat T lymphocytes (20), chorion-derived mesenchymal stem cells (14), activated mouse microglia (23), myeloid-derived suppressor cells (9), breast cancer (25) and glioblastoma multiforme cell lines (38).

Here, we evaluated the effects of H_V_1 inhibition by ClGBI, a guanidinium-containing compound, on the viability of polarized macrophages. Guanidinium-based H_V_1 blockers were described based on a seminal study demonstrating that substituting an arginine for the native asparagine at a residue localized at the narrowest part of the channel exerts deleterious effects on proton conduction. An identical blocking mechanism was found when applying soluble guanidinium ions intracellularly (3). When analyzing potential guanidine derivative inhibitors with different chemical modifications, ClGBI emerged as a compound capable of reaching its intracellular binding site from the extracellular side of the membrane and inhibiting the channel with relatively high affinity (24). Subsequently, ClGBI has been the most extensively used for inhibiting H_V_1 and investigate its function and biological relevance (8, 9, 13, 14, 20–23, 25). Here, we found that the H_V_1 inhibitor ClGBI dose-dependently reduced the viability of THP-1-derived macrophages with M0, M1 and M2 phenotypes as well (**Figure 2**), leading to death of most cells at concentrations widely applied in cellular studies examining the biological functions of the channel (8, 9, 13, 14, 21). However, a recent study examining the selectivity of ClGBI found that this compound also affects the function of various voltage-gated potassium and sodium channels such as K_V_1.1, K_V_1.3, K_V_1.4, K_V_1.5, K_V_10.1, K_V_11.1, Na_V_1.4 and Na_V_1.5 (77). This raises the question whether an H_V_1 inhibition indeed mediates ClGBI effects on macrophage viability, particularly, since these channels play important roles in the regulation of phagocytosis, migration and inflammatory cytokine release in macrophages (94, 95). Several lines of evidence support the H_V_1-mediated ClGBI effects and argue against its non-specific actions. i) First, blockers of relevant ion channels expressed in macrophages did not notably modify cell viabilities in our experiments at concentrations typically used in functional assays (**Figure 3**). ii) Second, Zn^2+^, another H_V_1 inhibitor (1), similarly compromised the viability of polarized macrophages (**Figure 3**). iii) Third, the extent of ClGBI effects correlated well with the expression level of H_V_1. Namely, M1 macrophages characterized by the highest H_V_1 abundance were the most sensitive to ClGBI, while M2 cells having lower expression displayed the lowest sensitivity (**Figures 2**, **4**). The higher abundance and importance of H_V_1 in M1 macrophages is consistent with previous studies. For example, it has been shown previously that a 24-h or 48-h treatment with the M1 cytokine TNF-α significantly increased the expression of proton currents in mouse bone marrow-derived macrophages (48). GM-CSF, another M1 cytokine, also enhanced H_V_1 expression in a STAT3- and STAT5-dependent manner in these cells (51). In accordance, LPS and IFN-γ activate NADPH oxidase (96, 97), which may in turn make the cell physiology more dependent on proton flux that is mainly provided by H_V_1 presumably leading to an increased expression of the channel in M1 cells and their augmented ClGBI sensitivity. Furthermore, in human peripheral blood monocyte-derived macrophages, H_V_1 inhibition by Zn^2+^ eliminated pH oscillations and led to sustained alkalinization of phagosomes of M1 macrophages, while in M2 cells it only exerted little effect on the fast development of phagosomal acidification (49). In keeping with the weaker ClGBI effects found in our study, IL-4 was recently shown to modulate Zn^2+^ homeostasis by triggering metallothionein- and Zn exporter-dependent increases in the labile Zn^2+^ pool, and presumably lower resulting H_V_1 conductance, in mouse bone marrow-derived macrophages (81). iv) Fourth, ClGBI-induced and H_V_1 inhibition-mediated toxicity was accompanied by an extensive pH dysregulation in the cells as discussed below.

Maintaining intracellular pH within the physiological range is essential for practically all cellular functions. H_V_1 is crucial in this aspect by providing a fast and efficient proton efflux during processes acutely acidifying the cytoplasm such as respiratory burst of immune cells or the Warburg effect of malignant cells (98). Accordingly, the deficiency or inhibition of the channel was previously shown to induce acidification in Jurkat T lymphocytes (20), isolated resting and activated mouse T lymphocytes (10), human sperm (13), canine ventricular myocytes (8), mouse dorsal root ganglion neurons (18), mouse and human microglial cells (23), breast cancer (25, 35, 36), colorectal carcinoma (37) and glioblastoma multiforme cells (38). The abnormal acidification induced by blocking H_V_1 was associated with compromised cell viability in most cases. However, such a connection has not been examined previously in macrophages. Here, we observed that the ClGBI-induced compromised viability was associated with significant acidification of the cytoplasm (**Figure 5**) in all examined macrophage classes with the largest effects observed in M1 cells that were already more acidic even in the absence of treatment when compared to M0 and M2 cells. In response to 100 µM ClGBI, the acidic pH shift, which was approximately 0.90, 0.54 and 0.48 pH units in M1, M0 and M2 cells, respectively, after 18 h treatment, can be considered large enough to substantially change cellular processes eventually culminating in cell death. Furthermore, such cytoplasmic alterations are expected to influence pH regulation of cellular compartments as well. While sporadic studies already proposed the expression and physiological roles of H_V_1 in phagosomes of macrophages (49, 50), the effects of channel inhibition have not been tested yet on lysosomes that represent important components of various cell death pathways (82, 83). In our measurements, we found large-scale time-dependent significant alkalinization of the lysosomes in response to a H_V_1 block, which reached a pH shift of 1.18, 0.78 and 0.73 in M1, M0 and M2 macrophages, respectively, in response to an 18-h application of 100 µM ClGBI (**Figure 6**), which is in accordance with previous reports on apoptosis-related alterations of the lysosomal pH in other cell types. The molecular mechanisms relating cytoplasmic and lysosomal pH alterations are very complex and incompletely understood (82, 99), and their elucidation is beyond the scope of the current manuscript. Nevertheless, H_V_1 channels are expected to provide a contribution and our findings altogether point at large-scale pH alterations throughout the cells in response to ClGBI-mediated H_V_1 inhibition.

Elevations in membrane ceramide levels can represent the link between pH dysregulation and cell death induced by blocking H_V_1 channels. Under resting conditions, ceramides are scarcely found in the cell membrane and constitute only <1% of total membrane lipids. However, stress stimuli substantially increase their abundance resulting in the formation of ceramide-enriched membrane platforms that facilitate signaling pathways participating in cell death processes including apoptosis, necroptosis, autophagy and ER stress and cell cycle arrest. Such mechanisms were shown to mediate cytotoxic effects of TNFα, ionizing radiation and various chemotherapeutic agents such as etoposide, cisplatin, daunorubicin, or gemcitabine plus doxorubicin combination. Ceramide overproduction occurs due to an imbalance of ceramide production and degradation mainly resulting from the activation of *de novo* ceramide synthesis involving serine palmitoyltransferase, or the degradation of sphingomyelin by neutral or acid sphingomyelinase (55–57). Since the activities of these enzymes are strongly pH-dependent (58–60), we hypothesized that ceramide overproduction resulting from the pH dysregulation-induced enzyme activity imbalance is an important factor leading to cell death in response to an H_V_1 block. Supporting this assumption, we observed time-dependent elevations in membrane ceramide levels in response to ClGBI (**Figure 7**). Interestingly, we also found already higher ceramide levels in M1 macrophages when compared to M0 and M2 cells even in the absence of a ClGBI treatment, which can result from an overexpression and activation of neutral and, in particular, acidic sphingomyelinase that are typical features of classical M1 polarization (100–102). The increased ceramide abundance can in turn elevate the propensity of M1 macrophages for ceramide overproduction and thus contribute to the higher sensitivity of these cells to the H_V_1 block-induced reduced viability. In addition, to unequivocally demonstrate the role of the ceramide level elevation, we tested the potential contribution of the ceramide producing enzymes using specific inhibitors, myriocin to reduce the activity serine palmitoyltransferase (84), GW4869 to block neutral sphingomyelinase (85), and ARC39 to inhibit acid sphingomyelinase (86). From among the tested inhibitors, ARC39 was able to impressively alleviate ClGBI-induced cell death, decreasing the effect of the H_V_1 blocker by 50-70% (**Figure 8**). Furthermore, ARC39 significantly attenuated elevations in plasma membrane ceramide levels in response to ClGBI, mainly in M1 cells. Altogether, our results imply that an H_V_1 inhibition compromises cell viability in a polarization-dependent manner, which results from a complex cellular pH dysregulation and ceramide accumulation that solidly depends on the activity of acid sphingomyelinase.

In summary, our study demonstrates for the first time that an inhibition of H_V_1 channels and the consequent complex cellular pH dysregulation compromises the viability of human polarized macrophages, most efficiently that of M1 cells, which is casually linked to ceramide overproduction that mainly depends on the activity of acid sphingomyelinase (**Figure 9**). Given that pH dysregulation and the resulting ceramide accumulation are expected to be general responses to H_V_1 inhibition, the reduced cell viability has to be taken into account when interpreting results of *in vitro* functional assays utilizing H_V_1 blockers. The medical relevance of our findings stems from the fact that H_V_1 inhibitors show great promise in various pathological conditions linked to ROS overproduction and associated inflammatory immune activation, and abnormal proliferation. In microglia of the nervous system, the H_V_1-mediated proton efflux is essential to counterbalance the acidosis arising from the charge transfer activity of NADPH oxidase producing ROS. Hence, a sustained ROS production is enabled by H_V_1 conductance, which plays crucial roles in neuroinflammation and consequent neuronal loss (11). Accordingly, in mouse disease models, genetic H_V_1 deficiency prevented neuronal death, brain damage and motor deficits in ischemic stroke (27, 28), carotid artery stenosis-induced white matter injury (103), demyelination in cuprizone- or lysophosphatidylcholine-induced multiple sclerosis models (29, 30), and neuronal apoptosis and pyroptosis, functional loss and pain hypersensitivity in traumatic brain and spinal cord injury models (31–34). Furthermore, a pharmacological inhibition of peripheral sensory neurons in dorsal root ganglia attenuated inflammatory pain and morphine-induced analgesic tolerance and hyperalgesia (18). These studies imply the beneficial applicability of H_V_1 inhibitors in neuroinflammation-associated pathological conditions. Considering the functional similarities between microglia and macrophages, the molecular mechanism of H_V_1 block-induced compromised viability outlined in this manuscript can contribute to the intended effects in these examples. However, potential off-target adverse effects involving impaired macrophage functions should be taken into account when applying H_V_1 blockers in these indications in the future. Malignant diseases comprise another group of disorders for the potential utilization of H_V_1 inhibitors. Rapidly proliferating neoplastic tumor cells are generally slightly depolarized and acidic due to a characteristic metabolic shift often referred to as the Warburg effect. Moreover, these cells typically overexpress the H_V_1 channel, which results in a highly beneficial efficient proton extrusion pathway. This provides a protective mechanism against intracellular acidosis and creates an acidic extracellular microenvironment that favors the degradation of the extracellular matrix facilitating invasion and contributes to an evasion from the immune surveillance (98). Consistently, H_V_1 overexpression is associated with disease severity and poor prognosis in breast cancer (36) and colorectal carcinoma (37). Furthermore, an siRNA-induced downregulation of H_V_1 inhibited proliferation, migration and invasion of both breast cancer, colorectal carcinoma and myeloid sarcoma cell lines, and a genetic H_V_1 deficiency delayed their growths in mouse tumor xenografts (15, 35–37). The pharmacological inhibition of the channel also seems promising as Zn^2+^ treatment reduced survival and migration of human glioblastoma multiforme cells (38) and ClGBI decreased cell viability in both monolayer and three-dimensional multicellular spheroid cultures of breast cancer cells (25). On the other hand, according to our results, an H_V_1 inhibition may also reduce the viability of polarized macrophages, mainly that of M1 cells with typical anti-tumor activities, and thereby favoring the predominant intratumoral appearance of M2-like tumor-associated macrophages, culminating in an M1-M2 imbalance, which may contribute to the immune evasion of neoplastic cells. These potential effects should be tested in the future, and may point to the need for a tailored selective tumor cell targeting approach for the application of H_V_1 blockers. Altogether, our findings imply that H_V_1 inhibitors can compromise the viability of polarized macrophages, which has to be taken into account both for their intended and adverse effects.

## Data Availability

The original contributions presented in the study are included in the article/**Supplementary Material**. Further inquiries can be directed to the corresponding author.
