# Supplementary material for: Inhibition of the HV1 voltage-gated proton channel compromises the viability of human polarized macrophages in a polarization- and ceramide-dependent manner
Source: Front Immunol. 2024 Dec 17;15:1487578. doi: 10.3389/fimmu.2024.1487578 (PMC11685079; doi:10.3389/fimmu.2024.1487578)
Supplement: Supplementary file 1 [file DataSheet1.docx]

Supplementary Material

# Supplementary Figures

**
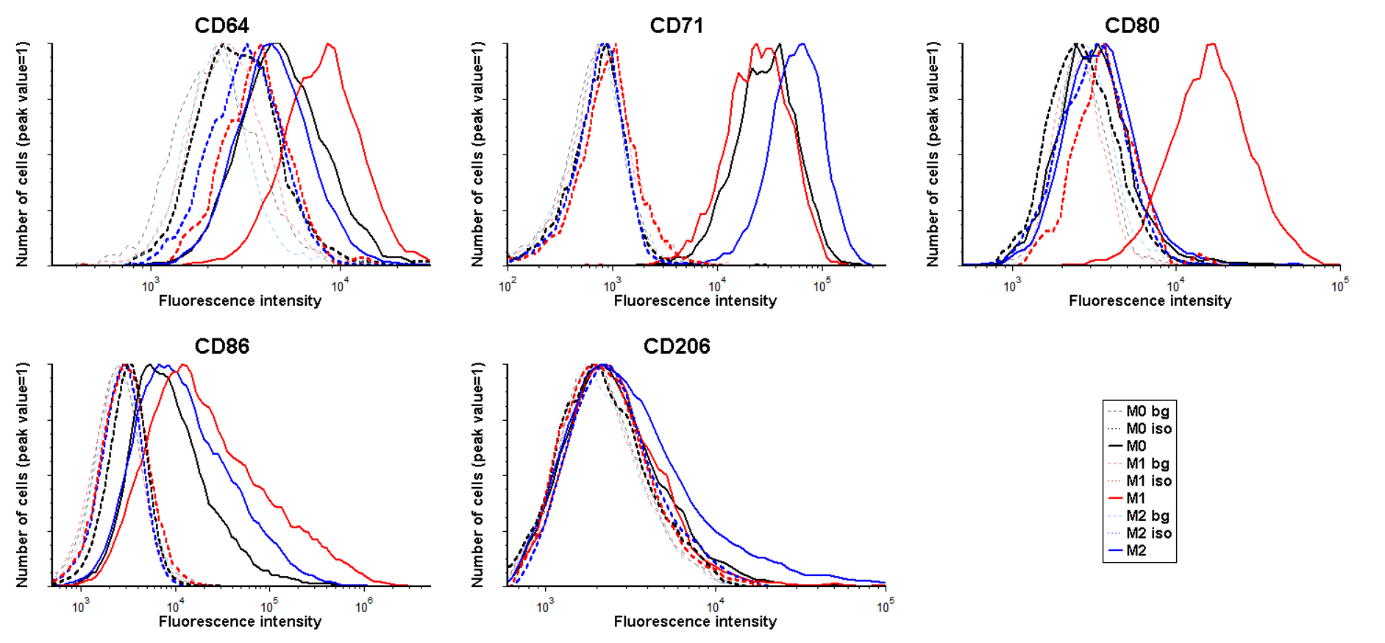
**

**Supplementary Figure 1.** Flow cytometric histograms of the expression of macrophage differentiation and polarization markers. THP-1 cells were differentiated into macrophages for 24 h by 10 ng/ml PMA, which was followed by a 24-h resting period in the absence of PMA. The differentiated M0 macrophages were subsequently polarized for 48 h into classical M1 macrophages with 100 ng/ml LPS plus 20 ng/ml IFN-γ, or M2 macrophages using 20 ng/ml IL-4 plus 20 ng/ml IL-13. After accutase-mediated detachment and Fc receptor blocking, cells were labeled with fluorophore-conjugated antibodies. As controls to examine the nonspecific binding of antibodies, cells were labeled with FITC-, Pe-Cy5-, or PE-conjugated Mouse IgG1 kappa Isotype Control, or Alexa Fluor 647-conjugated Mouse IgG2b kappa Isotype Control antibodies. Representative histograms of at least 10,000 individual cells of normal morphology per sample demonstrate the extents of cell surface CD marker expression when compared to unlabeled cells (thin dashed lines, ‘bg’) and those labeled with isotype controls (thick dashed lines, ‘iso’).


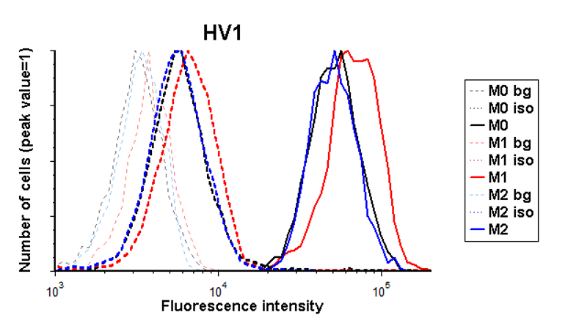


**Supplementary Figure 2.** Flow cytometric detection of the expression of H_V_1 in macrophages. THP-1 cells were differentiated into macrophages for 24 h by 10 ng/ml PMA, which was followed by a 24-h resting period in the absence of PMA. The differentiated M0 macrophages were subsequently polarized for 24 h into classical M1 macrophages with 100 ng/ml LPS plus 20 ng/ml IFN-γ, or M2 macrophages using 20 ng/ml IL-4 plus 20 ng/ml IL-13. After accutase-mediated detachment, fixation, permeabilization and Fc receptor blocking, the H_V_1 expression of differentiated and polarized macrophages was quantified using indirect immunofluorescence labeling and flow cytometry. In control experiments to examine the nonspecific binding of antibodies, cells were labeled with unconjugated Rabbit IgG Isotype Control primary antibodies. Representative histograms of at least 10,000 individual cells of normal morphology per sample demonstrate the extents of labeling when compared to cells labeled with secondary antibody only (thin dashed lines, ‘bg’) and those labeled with isotype control primary antibodies and the same secondary antibodies (thick dashed lines, ‘iso’).


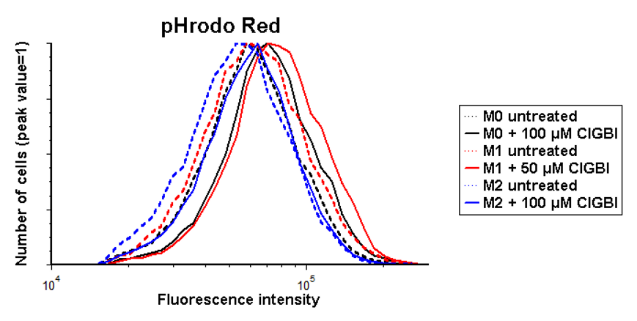


**Supplementary Figure 3.** Determination of cytoplasmic pH by flow cytometry. THP-1 cells were differentiated into macrophages for 24 h by 10 ng/ml PMA, which was followed by a 24-h resting period in the absence of PMA. The differentiated M0 macrophages were subsequently polarized for 24 h into classical M1 macrophages with 100 ng/ml LPS plus 20 ng/ml IFN-γ, or M2 macrophages using 20 ng/ml IL-4 plus 20 ng/ml IL-13. In the last 30 min of incubation, the cytoplasmic pH indicator pHrodo Red AM was further added to cells. After accutase-mediated detachment, the fluorescence intensity of individual cells was measured using flow cytometry. Corresponding pH values of individual cells were subsequently interpolated from the calibration curve determined based on calibration samples incubated with valinomycin and nigericin dissolved into cellular calibration pH buffers for Figure 5. Representative histograms of at least 10,000 individual cells of normal morphology per sample demonstrate changes in the fluorescence intensity of the pH indicator in response to ClGBI when compared to untreated cells (dashed lines).


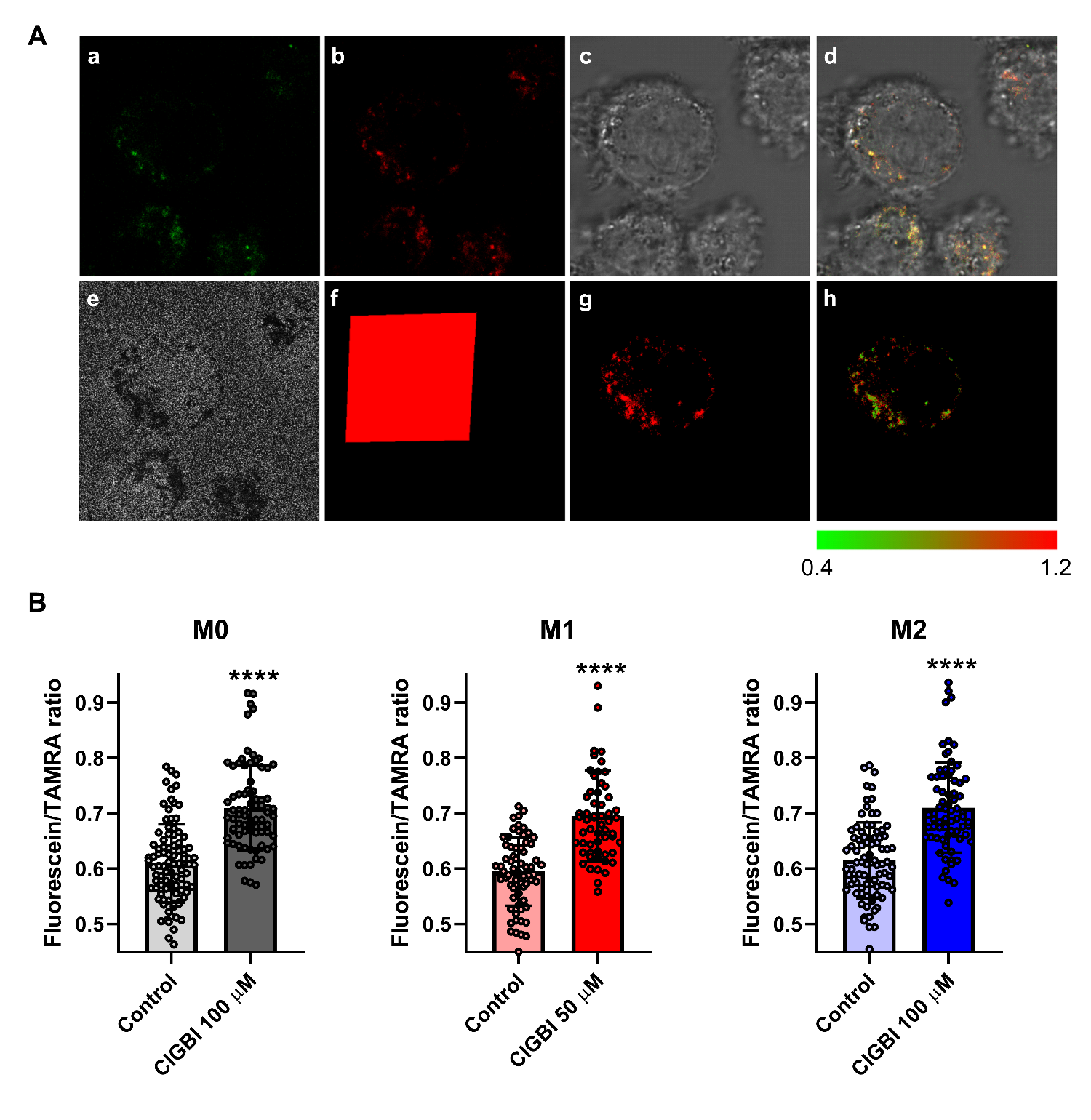


**Supplementary Figure 4.** Determination of lysosomal pH by confocal microscopy. (A) THP-1 cells were differentiated into macrophages for 24 h by 10 ng/ml PMA, which was followed by a 24-h resting period in the absence of PMA. The differentiated M0 macrophages were subsequently polarized for 24 h into classical M1 macrophages with 100 ng/ml LPS plus 20 ng/ml IFN-γ, or M2 macrophages using 20 ng/ml IL-4 plus 20 ng/ml IL-13. Cells were subsequently treated for 18 h with 50 or 100 µM ClGBI. 3 hours before the measurement time, 70,000 MW, anionic dextrane conjugated to pH-sensitive fluorescein and pH-insensitive tetramethylrhodamine (TAMRA) was added to cells for 1 h, which was followed by a chase of 2 h. Then, images were taken at the midplane of cells using a confocal microscope at channels corresponding to the fluorescein (a) and TAMRA fluorophores (b). Representative transmission (c) and overlay (d) images are also shown in the panel. During quantitative image analysis, after calculating the fluorescein/TAMRA fluorescence intensity ratio values for each pixel (e), a region of interest containing an individual cell was manually selected (f) and lysosomal pixels were identified based on their TAMRA fluorescence intensity above a threshold value (g). The calculated fluorescein/TAMRA fluorescence intensity ratios of individual lysosomal pixels are displayed for a representative cell on a color scale (h). (B) Subsequently, the average fluorescein/TAMRA fluorescence intensity ratio values positively correlating with the value of lysosomal pH were calculated for each individual cell using data of pixels corresponding to lysosomes. pH values were subsequently interpolated from the calibration curve determined based on calibration samples incubated with valinomycin, nigericin and bafilomycin dissolved into cellular calibration pH buffers for Figure 6. The fluorescein/TAMRA ratios obtained from n = 60-80 individual cells obtained from five independent experiments (± SEM) are plotted in the figure. Asterisks indicate significant differences compared to untreated control samples (*p < 0.05, **p < 0.01, ***p < 0.001, ****p < 0.0001, unpaired t test).


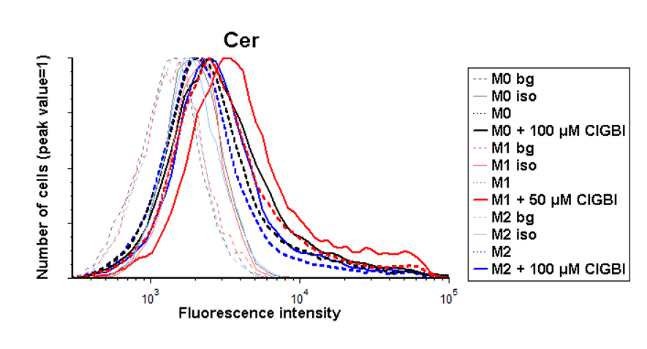


**Supplementary Figure 5.** Quantification of ceramide levels by flow cytometry. THP-1 cells were differentiated into macrophages for 24 h by 10 ng/ml PMA, which was followed by a 24-h resting period in the absence of PMA. The differentiated M0 macrophages were subsequently polarized for 24 h into classical M1 macrophages with 100 ng/ml LPS plus 20 ng/ml IFN-γ, or M2 macrophages using 20 ng/ml IL-4 plus 20 ng/ml IL-13. Cells were subsequently treated for 18 h with 50 or 100 µM ClGBI. After accutase-mediated detachment and Fc receptor blocking, cells were labeled with anti-ceramide antibodies followed by AlexaFluor647-conjugated goat anti-mouse IgM antibodies. In control experiments to examine the nonspecific binding of antibodies, cells were labeled with unconjugated Mouse IgM Isotype Control primary antibodies. Fluorescence intensities of individual cells were subsequently measured using flow cytometry and the representative histograms of at least 10,000 individual cells of normal morphology per sample demonstrate changes in the fluorescence intensity in response to ClGBI when compared to untreated labeled cells (thick dashed lines), cells labeled with isotype control primary antibodies and the same secondary antibodies (thin lines, ‘iso’), and unlabeled cells (thin dashed lines, ‘bg’).
